# Sex-specific changes in gene expression in response to estrogen pollution around the onset of sex differentiation in grayling (Salmonidae)

**DOI:** 10.1186/s12864-019-5955-z

**Published:** 2019-07-15

**Authors:** Oliver M. Selmoni, Diane Maitre, Julien Roux, Laetitia G. E. Wilkins, Lucas Marques da Cunha, Etienne L. M. Vermeirssen, Susanne Knörr, Marc Robinson-Rechavi, Claus Wedekind

**Affiliations:** 1 Department of Ecology and Evolution Biophore, University of Lausanne, Lausanne, Switzerland; 2 Swiss Institute of Bioinformatics, Lausanne, Switzerland; 3 Swiss Centre for Applied Ecotoxicology Eawag-EPFL, Dübendorf, Switzerland; 4 Aquatic Ecology and Toxicology Group Center of Organismic Studies, University of Heidelberg, Heidelberg, Germany; 5 Present Address: Swiss Federal Institute of Technology (EPFL), 1015 Lausanne, Switzerland; 6 Present Address: Department of Biomedicine, University of Basel, 4031 Basel, Switzerland; 7 Present Address: Department of Environmental Sciences, Policy and Management, University of California, Berkeley, CA 94720 USA

## Abstract

The synthetic 17α-ethinylestradiol (EE2) is a common estrogenic pollutant that has been suspected to affect the demography of river-dwelling salmonids. One possibility is that exposure to EE2 tips the balance during initial steps of sex differentiation, so that male genotypes show female-specific gene expression and gonad formation. Here we study EE2 effects on gene expression around the onset of sex differentiation in a population of European grayling (*Thymallus thymallus*) that suffers from sex ratio distortions. We exposed singly-raised embryos to one dose of 1 ng/L EE2, studied gene expression 10 days before hatching, at the day of hatching, and around the end of the yolk-sac stage, and related it to genetic sex (sdY genotype). We found that exposure to EE2 affects expression of a large number of genes, especially around hatching. These effects were strongly sex-dependent. We then raised fish for several months after hatching and found no evidence of sex reversal in the EE2-exposed fish. We conclude that ecologically relevant (i.e. low) levels of EE2 pollution do not cause sex reversal by simply tipping the balance at early stages of sex differentiation, but that they interfere with sex-specific gene expression.

**Electronic supplementary material:**

The online version of this article (10.1186/s12864-019-5955-z) contains supplementary material, which is available to authorized users.

## Background

Endocrine-disrupting chemicals are common pollutants that typically enter the environment after wastewater treatment. One of the most potent of these pollutants is the synthetic 17-alpha-ethinylestradiol (EE2) that is used in oral contraceptives and hormone replacement therapies, and that is more stable and persistent than the natural estrogen it mimics [1]. EE2 concentrations of 1 ng/L and higher have been found in river or lake surface waters [2], in lake sediments [3], and even in groundwater [4]. Concentrations around 1 ng/L therefore have ecological relevance.

Exposure to 1 or a few ng/L EE2 can be damaging to fish at various developmental stages. Embryos and early larvae can suffer from increased mortality, reduced growth, or malformations when exposed to EE2 [5–7]. In juveniles and adults, exposure to EE2 can affect the response to infection [8], increase the susceptibility to other pollutants [9], generally reduce growth and fertility [9, 10], and can even induce transgenerational effects on behavior and fertility in F1 [11] and F2 progeny [12]. Studies with experimental populations kept in 1,100 L ponds revealed population declines at concentrations of 1 ng/L EE2 [13]. Long-term, whole-lake experiments revealed significant ecosystem changes after experimental addition of 5–6 ng/L EE2: local populations of small fish declined (one species, the fathead minnow *Pimephalus promelas*, nearly disappeared), average body conditions of other fish, including top predators, declined significantly, and the prevalence of some zooplankton and insect species increased, possibly as a consequence of the reduced abundance of fish that prey on them [14]. As a result of these observations and associated risk analyses, the European Union recently proposed an annual average environmental quality standard of < 35 pg/L [15].

Experimental exposure to EE2 is associated with significant down- and up-regulation of various physiological pathways in fish. For example, juvenile sticklebacks (*Gasterosteus aculeatus*) exposed to 35–40 ng/L of EE2 showed reduced expression in testis for genes related to steroid biosynthesis (e.g., cytochrome P450 11A1 and 17A1, 3 beta- and steroid delta-isomerase 2) and increased expression in genes associated to epidermal growth (e.g., epidermal growth factor receptor) and xenobiotic metabolism (e.g., fms-related tyrosine kinase 4) [16, 17]. Juvenile coho salmon (*Oncorhynchus kisutch*) exposed to 2 or 10 ng/L of EE2 showed altered expression of genes linked to sexual-development and reproductive function: hepatic vitellogenin and pituitary luteinizing hormone were up-regulated, while pituitary follicle-stimulating hormone was down-regulated [16, 17]. Some of these effects on gene expression may be linked to the toxic effects of EE2 observed in juveniles and adults. However, it is likely that EE2 effects on gene expression depend on life history and on the developmental stage of an individual, i.e., on the timing of some physiological pathways in the organism. One important physiological pathway in this context is sex determination and gonad formation.

Sex determination is probably best seen as a threshold trait, with few processes that occur early in development determining the cascade of processes of gonad development [18]. In amphibians and fishes, these early processes can be very labile, i.e., potentially modifiable by external factors, even if they often have a clear genetic basis [19]. Among these external factors that interfere with these early steps of sex differentiation are temperature or endocrine disrupting chemicals such as aromatizable androgens [20, 21] or EE2 [19, 22] and other estrogens [22]. EE2-induced sex reversals are sometimes but not always observed [19, 22]. In salmonids, sex reversal can be induced by immersion of eyed eggs or larvae in high doses of EE2 (≥ 400 μg/L) (e.g. [23–25]). However, little is known about the effects of ecologically relevant concentrations of EE2 (i.e., around 1 ng/L or less) on gene expression at early stages of sex differentiation and on subsequent gonad formation.

Aside divergences in gonadal development, there are other fundamental differences between male and female development in fishes. These include, for example, average growth and timing of maturation [26], habitat use [27] or susceptibility to various stressors including infections [28, 29]. In this regard, it remains unclear whether long-term differences should also be expected between the genetic sexes upon the effect of EE2 [30, 31]. Such questions can be studied if reliable sex-linked genetic markers are available for a given study species.

Here we study the sex-specific gene expression and gonad development in grayling (*Thymallus thymallus*), a river-dwelling salmonid that is likely to be exposed to EE2 pollution when its habitat receives treated wastewater [32]. Yano et al. [33] established sex-linked genetic markers that can be used to determine the genetic sex of many salmonids. These markers could be successfully verified in over 100 phenotypically sexed adult grayling sampled from our study population [26]. Genetic sexing of juvenile grayling revealed that sex differentiation takes place during the first 6 months after hatching and goes through a male stage in both sexes [26]. This makes the grayling a rare example of a so-called “undifferentiated” gonochoristic species [34]. Genetic female grayling first develop testis tissues, followed by a testis-to-ovary stage (with perinuclear follicles loosely scattered within testicular tissue), before ovaries are developed, consisting of perinuclear follicles and oogonia [26]. Moreover, sex differentiation is delayed in male grayling who instead grow faster than females during the first months [26].

We use genetic sexing to study sex-specific effects of a low and hence ecologically relevant concentration of EE2 on gene expression at the onset of sex differentiation. Maitre et al. [26] found large differences between genetic sexes of grayling at the gene expression level (in heads) around the time of hatching from eggs, while gene expression (whole embryos) did not seem to differ significantly between the sexes at late embryogenesis. Their findings suggest that the physiological cascade of sex differentiation starts during embryogenesis and before hatching, which is consistent with patterns observed in other salmonids [35, 36]. We therefore study the interaction between EE2 and genetic sex on gene expression in embryos, hatchlings, and juveniles. Within-family comparisons are used to minimize potential effects of genetic variation. Possible effects of EE2 on gonad development are then studied histologically on samples taken over a period of several months of juvenile development.

## Methods

### Experimental breeding, rearing, and treatment

Ten males and 8 females were sampled from a captive breeding stock and stripped for their gametes. These fish are F1 of the wild population that spawns in the River Aare in the city of Thun, Switzerland [37]. Their gametes were used in two full-factorial breeding blocks. For each breeding block, four females were crossed in vitro with five males, i.e., 40 (2x4x5) different sibgroups were produced (Additional file 1: Figure S1). After egg hardening for 2 h, the fertilized eggs were transported to the laboratory where they were washed and distributed to 24-well plates with low-evaporation lids (Falcon, Becton-Dickinson), following the methods of von Siebenthal et al. [38]. In total, 10,789 eggs (range 184–352 per sibgroup) were distributed (one egg per well). The wells had been filled with 1.8 mL chemically standardized water [39] that had been oxygenated and temperated before use. Eggs were considered fertilized if embryos were visible 14 dpf (days post fertilization). Overall fertilization rate turned out to be 69.8%. The embryos were then used for several parallel studies: to compare the effects of EE2 and other environmental stressors on embryo development in different salmonid species (Marques da Cunha et al., in preparation), and to study sex differentiation in grayling [26].

The embryos used for the present study (Additional file 1: Figure S1) were incubated in a climate chamber set at high humidity to further minimize evaporation, and at 7 °C for the first 27 dpf. At 27 dpf, the temperature of the climate chamber was raised to 10 °C, and to 11.5 °C 1 day later to induce and synchronize hatching and hence to increase comparability across all the samples of gene expression at hatching time. The temperatures we used correspond well to the mean temperatures that the embryos would experience at the natural spawning place [37]. Temperature changes of 3–5 °C within a day are frequently observed at the natural spawning place [37, 40]. These ecologically relevant mean temperatures, and the induced temperature changes, do not influence sex determination [40].

**Fig. 1:**
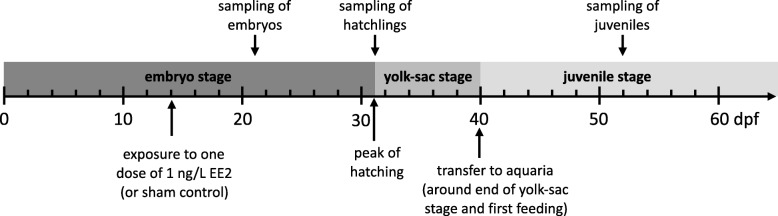
Timing of the treatments and of the sampling for transcriptomics analyses relative to the developmental stages (dpf = days post fertilization)

Figure 1 outlines the timing of the treatment and the sampling (see Additional file 1: Figure S1 for further details). We left the freshly distributed embryos undisturbed until 14 dpf in order to minimize mortality due to handling during the very first stages of embryogenesis. We then largely followed protocols of previous studies, i.e., embryos were exposed either to one dose of 1 ng/L EE2 (by adding 0.2 mL water with a concentration of 10 ng/L EE2, see Brazzola et al. [6] and Marques da Cunha et al. [7] for details) or sham-treated (“control”, i.e., only adding 0.2 mL standardized water). We used this concentration of EE2 because it appears ecologically relevant [2–4]. Additional treatments with *Pseudomonas fluorescens or P. fluorescens* plus one dose of 1 ng/L EE2 were done on further embryos in the course of a parallel study (Marques da Cunha et al. in preparation). We did not sample *P. fluorescens-*treated individuals for the gene expression analyses, but about half of the juveniles that were sampled for the histological analyses were *P. fluorescens-*treated. This allowed us to test for possible interaction effects between EE2 and *P. fluorescens* on gonad formation.

Marques da Cunha et al. [7] used a similar protocol on embryos of brown trout (*Salmo trutta*; another salmonid), i.e., they also exposed early embryos to one dose of 1 ng/L EE2 (at the slightly colder rearing temperature of 4.6 °C) and found the EE2-concentrations in wells with embryos to decline to close to zero within 4 weeks while remaining largely stable in wells without embryos. We therefore assume that the 2 pg EE2 that we added per 2 mL well were largely taken up by the embryos during the observational period.

Hatched larvae were raised in the well plates until 40 dpf, i.e., until several days after hatching and around the end of the yolk-sac stage, when about 660 individuals per treatment group were about equally distributed to two 200 L tanks each (Fig. 1, Additional file 1: Figure S1). Individuals assigned to transcriptome analysis (5 individuals per sibgroup and treatment) were separated in net cages within the aquaria that corresponded to their treatment (Additional file 1: Figure S1). The tanks were filled with lake water that had been pumped from Lake Geneva at 40 m depth. The physico-chemical parameters of freshly pumped water as determined in early July with a pHmeter744 (Metrohm, Switzerland) and a FireStingO2 (PyroScience, Denmark) were: temperature = 7.6 °C, pH = 7.7, oxidation-reduction potential (mV) = − 42, O_2_ = 10.46 mg/L). The tanks were filled several days before the fish were introduced, i.e., water temperature could adapt to the temperature in the climate room. The juveniles were first fed with live *Artemia* and copepods and later with dry food. For the EE2-treated groups (i.e. juveniles that had been exposed to EE2 during the embryo stage and were now exposed again during the juvenile stages), 200 ng EE2 were dissolved in 200 L tanks each to reach a starting concentration of 1 ng/L. Every 7 days from then on, 40 L per tank (i.e., 20%) were replaced with fresh lake water. In the EE2-treated groups, these 40 L were spiked with 40 mL of a 1 μg/L EE2 stock solution (i.e., 40 L at 1 ng/L EE2). Water samples (100 mL each) were then taken from each of the 4 EE2-treated tanks 1 h after this weekly water exchange (T_0_) and 7 days later, just before the next water exchange (T_7_). These water samples were immediately frozen and stored at − 20 °C protected from light. Four consecutive T_0_ and 4 consecutive T_7_ samples were each pooled per tank for later determination of EE2 concentrations, i.e., EE2 concentrations were determined for the 4-week intervals these pooled samples covered, starting 47 dpf, 75 dpf, 103 dpf, and 131 dpf, respectively. Fish were not analysed for EE2 concentration in their body.

### Histology

Histological examinations were used to study potential effects of EE2 on sex differentiation. In total 256 fish were randomly sampled 51, 79, 107, 135, 159–163 *dpf* (Additional file 1: Figure S1) *and* fixed in Davidson solution (AppliChem product No. A3200). Processing followed standard procedure with dehydration of the samples, embedding in paraffin, and processing of 4 μm thick serial sections of the gonads performed from ventrally through the whole body. Sections were stained with Mayer’s haematoxylin and eosin and cover slipped for conservation. Fish sections were analyzed by light microscopy. Gonads were categorized as “undifferentiated” (gonad consists entirely of undifferentiated cells), “testis” (spermatogonia and spermacytes), “testis-to-ovary” (perinuclear follicles scattered within testicular tissue), or “ovary” (perinuclear follicles and oogonia only). See Additional file 1: Figure S2 for representative examples of these four categories.

Of the 249 fish that could be successfully processed (seven were lost during handling for histology), all 101 fish sampled at the last two sampling periods (135 dpf and 159–163 dpf) were genetically sexed based on genomic DNA extracted from tissue samples and following the PCR protocol of Yano et al. [33] with slight modifications as explained in Maitre et al. [26] (we accidentally missed genetically sexing the first samples).

### Monitoring EE2 concentrations in the tanks

To quantify EE2 in the 200 L tanks, the water samples were thawed and filtered through glass fibre filters, their volume was set to 250 mL and the pH to 3. Four ng/L of EE2 D4 was added as internal standard and samples were enriched on LiChrolut EN / LiChrolut RP-C18 cartridges that had been conditioned with hexane, acetone, methanol and finally water (pH 3) [41]. After sample enrichment, cartridges were dried with nitrogen and eluted with acetone and methanol. Subsequently, solvents were changed to hexane/acetone 65:35 and extracts were passed over Chromabond Silica columns [42] and set to a volume of 0.25 mL. LC-MS/MS analysis was performed on an Agilent6495 Triple Quadrupole. An XBridge BEH C18 XP Column, 2.5 μm, 2.1 mm X 75 mm and an acetonitrile / water gradient was used for liquid chromatography followed with post-column addition of ammonium fluoride solution. EE2 was quantified by monitoring the mass transition of 295 to 269, the transition of 295 to 199 served as a qualifier (internal standard was quantified at the following transitions: 299 to 273 and 299 to 147). EE2 concentrations were calculated in a sample when the signal to noise ratio of the EE2 quantifier and qualifier peaks exceeded 10. The LOQ (limit of quantification) in the samples ranged from just below 0.1 ng/L at the start of the experiment to just above 0.2 ng/L towards the end of the experiment.

In the 200 L tanks, median EE2 concentrations were 0.33 ng/L at T_0_ and 0.11 ng/L at T_7_, corresponding to a median reduction of 66% of the EE2 dissolved in water over 7 days (see Additional file 1: Figure S3). We found no significant effects of sampling period on the EE2 measures at T_0_ (ANOVA, F_3_ = 1.20, *p* = 0.35) nor on the weekly reduction of EE2 in the tanks (F_3_ = 1.88, *p* = 0.19; excluding an unexplained outlier, see Additional file 1: Figure S3 for discussion).

Control aquaria were not spiked with EE2. However, it turned out that the 15 probes we analysed from sampling control aquaria showed unexpected EE2 concentrations of up to 12.8 ng/L, suggesting accidental contamination of probes. A comparison between EE2-treated and control individuals sampled from the 200 L tanks would therefore be based on the assumption that contamination happened after water sampling and that the control tanks had never been exposed to EE2. The observed contamination is indeed likely to have happened after sampling (see Additional file 1: Figure S3 for discussion). However, even if this were not true and the control tanks had been accidentally exposed to EE2, test for genotype-phenotype mismatches in the EE2-treated group are still valid.

### Gene expression analyses

For the gene expression analyses, we focused on five sibgroups sharing the same mother but differing for the identity of the father (Additional file 1: Figure S1). EE2-treated and controls from each sibgroup were sampled at three distinct time points (Fig. 1; Additional file 1: Figure S1). The first sampling of 12 embryos per family and treatment took place at 21 dpf, i.e., 9 days after treatment and well before hatching could be expected. Embryos were immediately transferred to RNAlater (Thermo Scientific, Reinach, Switzerland). The second sampling took place at the day of peak hatching for all treatment groups, i.e., 31 dpf (8 hatchlings per family and treatment). The third sampling took place at 52 dpf (5 juveniles per family and treatment). Hatchlings and juveniles were narcotized with 0.5 mL/L KoiMed (fishmed GmbH, Galmiz, CH) for 5 min and then decapitated. The heads were immediately transferred to RNAlater. All samples were stored at − 80 °C.

RNA was extracted using the QIAgen 96 RNeasy Universal Tissue Kit (QIAGEN, Hombrechtikon, Switzerland). Manufacturer instructions were followed except that centrifugation (Eppendorf 5804 R centrifuge with an A-2-DWP rotor; Eppendorf, Schönenbuch, Switzerland) was done twice as long at half the speed. Because the RNA extraction protocol did not include a DNase treatment, DNA traces inside the RNA samples were amplified to determine the sdY genotype [33] of each individual, i.e., the presence or absence of the male-specific Y-chromosome sequence of many salmonid fish, using the 18S gene as PCR internal control. The sdY genotype was determined either in a multiplex reaction used for samples with higher relative amount of DNA, or after a second PCR amplification in single reactions with half the amounts of the respective primers each for samples with low DNA content (see Maitre et al. [26] for a more detailed protocol). Based on the sdY genotype, one female and one male per family and treatment group (i.e. combination of family, treatment and time point) was haphazardly chosen for a total of 60 samples selected for transcriptomics analyses (in 2 of the treatment groups, two females or two males were used because only one sex could be found). Of note, we verified that the relative amount of DNA had no notable effect on the transcriptome analyses. For that, we measured the 260 nm/280 nm absorbance ratio (i.e. a proxy value for DNA contamination [43]) of every sample, and did not observe any particular association with RNA sequencing depth nor gene expression patterns (Additional file 1: Figure S4A and B, respectively).

The RNA extracts were provided for library preparation in an equimolar concentration of 6 ng/μL in 100 μL of volume. Fifty microliters (i.e., 300 ng of RNA) were each used for library preparation on a robot using the Truseq Stranded RNA protocol (Illumina, Part# 15026495 Rev. A). This protocol employs two steps of poly A selection required to purify total RNA from possible DNA contamination. Importantly, the mean 260/280 absorbance ratio of the 60 samples prior to library preparation was 2.093, showing therefore an already minimal level of DNA contamination. The libraries were then introduced in the Illumina sequencing platform (HiSeq 2500) for 100 cycles of multiplexed paired-end reads sequencing. The total 60 samples were sequenced in ten lanes (six samples per lane).

### Bioinformatics pipeline

The early processing of RNA-seq reads followed the pipeline described in Maitre et al. [26]. To summarize, reads were quality trimmed or filtered, resulting in a set of 60 RNA libraries with, on average, 2*40 millions of 80 bp reads each (standard deviation of 6 million reads). Next, reads from all libraries were pseudo-mapped onto the recently published Grayling genome-based transcriptome [34, 44] using Kallisto (version 0.42) [45]. Principle component analysis was performed on TMM-normalized [46] log2(count-per-million) values (CPM). Differential expression analysis was performed using the limma-voom Bioconductor package (version 3.26.3) [47, 48] with sample quality weights [49] on CPM values that were additionally cyclic loess normalized. In the linear model we considered developmental stage, sex and treatment as a combined variable (with 12 possible levels) and sib-group as an independent variable. A linear model was then fit for each gene, coefficients and standard errors were computed for all the contrasts of interest. Q-values [50] were calculated for each gene, and a threshold of q = 0.15 was used to call differentially expressed genes unless specified otherwise. Transcripts were annotated by referring to the annotation of the reference transcriptome and the associated genome. An enrichment analysis of gene ontology (GO) terms was performed on differentially expressed genes using the goseq Bioconductor package (version 1.22.0; [51]). Raw data of the analysis of RNA quality, PCR-based amplification of the locus associated with sex, RNA-sequencing reads quality are provided in Additional file 1: Table S7. Comparison of gene expression patterns between control individuals is described in Maitre et al. [26].

## Results

In total, the gene expression analysis involved 38,372 genes, which were almost all found expressed at the three sampling stages (sum of the pseudocounts per gene > 0 in 38,359, 38,370, and 38,366 genes in embryos, hatchlings, and juveniles, respectively).

### Differential gene expression

In order to test for sex-specific effects, we compared the changes in gene expression under EE2 treatment for individuals of the same sex at the same developmental stage (Table 1). Under EE2 treatment, at the embryo stage there was an altered expression of several hundred genes in genetic males (Table 1a, Additional file 1: Figure S5a and Table S1), but only of a few genes in genetic females (Table 1a, Additional file 1: Figure S5b).

**Table 1:** Number of genes that are differentially expressed (q < 0.15) in males and females of the different treatment groups (EE2-treated or control) tested at (a) embryo stage, (b) hatchling stage, and (c) juvenile stage at the onset of exogenous feeding

	Control females	EE2-exposed males	EE2-exposed females
a) Embryos			
Control males	10	383	369
Control females		2	15
b) Hatchlings			
Control males	21,190	1^a^	0
Control females		1	20,420
c) Juveniles			
Control males	466	4	5
Control females		2,986	9,979

^a^ 10,683 with q < 0.25, see Additional file 1: Figure S5

At hatching day, genetic males displayed no significantly altered expression at a false discovery rate (FDR) of 15%; yet there was a weak signal of expression change for more than 10,000 genes at 25% FDR (Table 1b, Additional file 1: Figure S5c and Table S2). Females, in contrast, displayed a net alteration in the expression of over 20,000 genes (15% FDR; Table 1b, Additional file 1: Figure S5d and Table S3).

At the first feeding stage the expression of only very few genes appeared altered in genetic males (Table 1c, Additional file 1: Figure S5e), whereas in genetic females around 10,000 genes were affected (Table 1c, Additional file 1: Figure S5f and Table S4).

In Table 2, the sex-specific alterations in gene expression are split according to the direction of the changes. At embryo stage, 149 genes were up-regulated under EE2 in males while 233 were down-regulated (15% FDR, Table 2). Around hatching, about 4,500 genes were up-regulated in EE2-treated genetic males while down-regulated in EE2-treated genetic females, and about 3,500 were down-regulated in EE2-treated genetic males while up-regulated in EE2-treated genetic females (25% FDR, Table 2). The remaining sex-specific reactions to the EE2 treatment were mainly up- or down-regulation in one sex while there was apparently no change in the other sex (Table 2). At juvenile stage, EE2 treated females had 6,090 genes up-regulated and 3,884 down-regulated. See Additional file 1: Tables S1-S4 for EE2 effects on up and down-regulation of gene expression in both, genetic males and females. Table 3 provides a summary interpretation of the gene expression analysis.

**Table 2:** Number of genes that were upregulated, i.e., had a positive log fold change of expression with q < 0.15 (UP), experienced no significant change in expression (NO), or were downregulated (DO) after exposure to EE2. At hatching the q threshold was set to q < 0.25, see text and Additional file 1: Figure S5

	Males UP	Males NO	Males DO	Total
a) Embryos				
Females UP	0	7	0	7
Females NO	149	37.599	233	37.981
Females DO	0	7	1	8
Total	149	37,613	234	37,996
b) Hatchlings				
Females UP	267	9,987	3,588	13,842
Females NO	1,275	8,040	977	10,292
Females DO	4,341	9,073	234	13,648
Total	5,883	27,100	4,799	37,782
c) Juveniles				
Females UP	3	6,090	0	6,093
Females NO	1	27,733	0	27,734
Females DO	0	3884	0	3,884
Total	4	37,707	0	37,711

**Table 3:** Summary interpretation of the differential gene expression analysis. The characterization of the biological processes relies on the gene ontology enrichment analysis of differentially expressed genes. Feminization and masculinization represent the situation where few genes (< 100) are detected as differentially expressed, under EE2 treatment, in comparison to control female or control male, respectively. See Additional file 1: Figure S4 and Tables S1-S5 for more detailed information

Developmental stage	Sex differences in gene expression ^a^	EE2-effects in males	EE2- effects in females
Embryos	Weak	~ 1% of the genes are affected, some related to the development of the nervous system.	None detected.
Hatchlings	Strong	Up to 18% of the genes are affected, enriched in association to muscle and connective tissues. Possible transient feminization.	Up to half of the genes are affected. Possible masculinization.
Juveniles	Strong	A few genes only are affected.	Up to 25% of the genes are affected, enriched in association to insulin metabolism and heart development. Possible masculinization persists.

^a^ Results from Maitre et al. [26]

We checked expression patterns of known sex-related genes and of genes associated to estrogen metabolism (Additional file 1: Table S5). Almost all of the changes in expression related to these genes were observed in EE2-treated females at hatching and at juvenile stage. The only gene of this list significantly changing in males is Cytochrome p450 1A1, which was upregulated in EE2-treated males at the juvenile stage. Out of the 68 genes in this list, 46 showed a change in gene expression under EE2. The most common pattern was a decrease of expression in females under EE2 at hatching (31 genes). In 10 of these cases the same genes were up-regulated in females under EE2 at the juvenile stage (for example, Cytochromes P450 1A1, Estrogen receptor beta). In the remaining 22 genes no significant effect was detected. These notably include aromatase cyp19a1, Estrogen receptor beta-1, and Epidermal growth factor receptor. Of note, cyp19a2 is not detected at all in our transcriptome data, consistent with only cyp19a1 being a brain aromatase in fish [52]. Increased expression in hatching females under EE2 was rare (8 genes, among them another copy of the Epidermal growth factor receptor). In 7 cases, changes in gene expression under EE2 were observed only in females at the juvenile stage, of which 6 decreased in expression (notably Cytochrome p450 1B1, Androgen receptor and another copy of the Epidermal growth factor receptor).

### Does EE2 treatment feminize males and masculinize females?

After focusing on sex-specific gene expression changes induced by EE2 treatment, we compared control males against EE2-treated genetic females and control females against EE2 treated genetic males (Table 1). The aim of this analysis was to investigate whether the EE2 treatment would feminize males, masculinize females, or increase the differences in gene expression between sexes. At the embryo stage, we found only two genes differentially expressed between EE2 treated genetic males and control females (Table 1a) and 369 genes between control males and EE2 treated genetic females (Table 1a). At hatching day, we found no differences in gene expression levels between control males and genetic females treated with EE2 (Table 1b) and only one gene differing between control females and EE2 treated genetic males (Table 1b). At first feeding stage, EE2 treated genetic males expressed around 3,000 genes differently in comparison to control females (Table 1c), while gene expression in control males differed in five genes only from the gene expression of EE2-treated genetic females (Table 1c). We do not expect any power difference in these tests compared to the previous within-sex tests for differential expression so there does appear to be transcriptome evidence of feminization of genetic males at hatching and of masculinization of genetic females at hatching and at juvenile stage.

### Gene ontology enrichment analysis

Additional file 1: Tables S1-S4 show the top 25 GO terms enriched in genes up- or down-regulated under EE2 at different stages. In most cases, these GO terms referred to broad molecular processes (for instance *G-protein coupled activity*, *chromatin*, *endopeptidase activity*, etc.). Some enriched terms were more specific. For instance, in males at the embryo stage, genes upregulated under EE2 were enriched for terms potentially related to the development of the nervous system such as *neuropeptide Y receptor activity*, *postsynaptic membrane*, and *electron carrier activity.* In males at hatching, GO terms enriched for genes altered under EE2 suggested changes in development of muscular tissues (*motor activity*, *myosin complex*) and of the connective tissue or dermis (*collagen trimer*, *keratin filament*). In females at the juvenile stage, GO terms associated to glycogen metabolism (*glycogen metabolic processes*, *insulin receptor signalling pathway*) and to *heart development* were enriched for genes down-regulated under EE2.

### Sex differentiation

Exposure to EE2 delayed the onset of morphological sex differentiation while exposure to *P. fluorescens* showed no effects (Additional file 1: Table S6 and Figure S6). Only testis tissue could be observed at the 2nd sampling (79 dpf), while the rate of ovarian tissue rose quickly to 70.8, 72.4%, then 75.0% over the 3rd, 4th, and 5th sampling periods, respectively. The rates of ovarian versus testis tissue did not differ between EE2-treated and controls (χ^2^ = 0.23, *p* = 0.63).

Genetic sexing of all 101 individuals of the 4th and 5th sample (135 dpf and 159–163 dpf) revealed a genetic sex ratio (i.e. male proportion) of 54.5% that did not deviate from equal sex ratio (χ^2^ = 0.8, d.f. = 1, *p* = 0.27). Equal sex ratios were therefore assumed for all earlier samples. At these two last sampling days, all genetic females except four showed ovarian tissue (ovaries or testis-to-ovaries). The four exceptions (two EE2-treated and two controls) showed testis tissue, i.e., no genetic female was undifferentiated at that these last sampling dates. In contrast, 44 of a total of 55 genetic males (80%) were still undifferentiated at that time, the remaining 11 showed testis tissues.

## Discussion

We tested and describe the effects of exposure to low, ecologically relevant, concentrations of EE2 on sex-specific gene expression in embryos and juveniles of grayling, a river-dwelling salmonid that is often exposed to this type of pollution [53]. From what is known about possible EE2 effects on fish in general, we expected that this common pollutant may (i) affect sex determination of grayling by influencing the few initial triggers that start the canalized developmental process of gonad formation, and (ii) be toxic to the embryos and juveniles because it interferes with different types of physiological processes, especially those that are endocrinologically regulated (see references cited in the Introduction). We therefore expected EE2 to have significant effects on gene expression at various developmental stages, and we indeed found such effects at all the developmental stages we studied here. However, we had no clear a priori expectancy about whether EE2 would also affect the genetic males and genetic females differently at any of these stages.

We started from the premise that sex in gonochoristic species is a threshold trait, i.e., a canalized developmental process that has one or few initial triggers [18]. In grayling, the initial trigger (or triggers) that determine phenotypic sex happen during embryogenesis well before hatching, since over 20,000 genes are already differentially expressed between genetic males and females at the day of hatching [26]. The few genes that Maitre et al. [26] found to be differentially expressed in genetic males and females at the embryo stage 10 days before hatching suggest that sex differentiation starts around then, i.e., at a time when the embryos had already been exposed to EE2 for several days in the present study. Of note, Maitre et al. [26] used a de novo transcriptome whereas here we mapped transcripts to a published genome [44], which is more reliable and more powerful (61% of reads mapped to the genome, vs. 52% to the de novo transcriptome). Thus, while numbers are slightly different between our Table 1 and Maitre et al. [26], the trends are consistent. There is weak evidence that a transcript of cytochrome P450 1d1 is already six-fold more expressed in females than males in embryos; most other known sex marker genes are only significantly differentially expressed at hatching [26].

One possible scenario is hence that EE2 could tip the balance at the early steps of sex differentiation so that all individuals follow the developmental process that leads to the female phenotype regardless of their sdY genotype; i.e., sex reversal of genetic males. If so, EE2 may not be expected to show strong sex-genotype specific effects on gene expression during later stages of sex differentiation. However, we found strong interactions between genetic sex and EE2 on gene expression. These sex-specific reactions to EE2 also depended on the developmental stages we studied. At the embryo stage, expression of only few genes seemed biased in genetic females, but gene expression in genetic males was already significantly affected, with about 400 genes up- or down-regulated under the influence of EE2. The outcome was somewhat reversed in juvenile heads: now only few genes of genetic males seemed to be affected by EE2, while over 9,000 genes were differentially expressed in genetic females. An even more pronounced effect of EE2 could be seen in heads at the day of hatching: over 20,000 genes showed differential expression, and about half of them were either up-regulated in genetic females and down-regulated in genetic males or down-regulated in genetic females and upregulated in genetic males.

The strong sex-specific responses to EE2 suggest that exposure to ecologically relevant concentrations of EE2 during embryogenesis did not simply tip the balance at early steps of sex differentiation, so that all individuals would become phenotypic females and would show similar patterns of gene expression from then on. Instead, our observations suggest that genetic sex largely determined phenotypic sex, and that EE2 then interfered with sex-specific gene expression, creating the strong sex-specific reactions to EE2 in the head. This conclusion is supported by the observation that at the low concentrations of EE2 commonly observed in natural rivers and streams, similar to those we used, there is little evidence for complete and population-wide sex reversal, even if natural populations sometimes show distorted sex ratios [54]. However, it is still possible that higher doses of EE2 can tip the balance at early steps of sex differentiation and thereby affect phenotypic sex. It would then be interesting to compare gene expression relative to genetic sex versus phenotypic sex, and to study more tissues.

The interaction between EE2 and genetic sex on gene expression suggests that exposure to EE2 is mostly interfering with the development of a phenotype that would correspond to the genotypic sex. It is possible that we missed sex reversal (genetic males developing ovaries), because we learned only during the course of the study that the grayling is a rare example (and probably even the only one so far) of an undifferentiated gonochorist that goes through an all-male stage before gonads differentiate into testes and ovaries [26]. Testis tissue in early juveniles can therefore neither be interpreted as evidence for normal development of a male phenotype nor as evidence for sex reversal in genetic females. However, by the end of the study, nearly all genetic females had developed ovarian tissue. This suggests that the rate of sex reversals is either low indeed, or that sex reversal would slow down gonad development so much that we would have missed many sex-reversed individuals within our observational window. We know of no examples or arguments in the literature that would support the latter possibility. Moreover, EE2-induced sex reversal would lead to mismatches between genotypes and phenotypes that can, on the long term, affect population demography [55]. Wedekind et al. [54] and Maitre et al. [26] specifically searched for such mismatches in a wild population of grayling and found none. The authors concluded that the distorted population sex ratios that have been observed in their study population are not due to environmental sex reversal (see also [40]) but more likely linked to sex-specific mortality. It remains to be tested whether sex-genotype specific reactions to endocrine-disrupting pollutants can contribute to sex-specific mortality in the wild.

Our gene expression analysis suggested that exposure to EE2 induces effects in the transcriptomes of the brain that could be interpreted as partial sex reversal: At the day of hatching, we did not find any significant difference between the gene expression patterns in the heads of EE2-treated males and control females. This apparently feminizing effect of EE2 seemed to cease before the (next sampled) juvenile stage. In contrast, gene expression in the heads of EE2-treated genetic females was alike the one of control males at both hatching and juvenile stage, as if exposure to EE2 induced partial masculinization. Estrogens are known to affect functions of the nervous system, including synapsis homeostasis [56], neurogenesis [57] and sexual differentiation [57–59]. In mammals, for instance, aromatizable androgens (e.g. testosterone) are converted into estrogen by brain aromatases to promote masculinization of brain [58]. In fish, however, the role of estrogen in the development of sexual behaviors is still poorly understood [60], and is more labile than in mammals. Effects of EE2 on male behavior have been observed in goldfish [61], while, to our knowledge, no study reported effect on females. In general, in fish aromatases are feminizing enzymes [62, 63]. But in fish brain, estrogen strongly up-regulates aromatases and differentiating male rainbow trout (*Oncorhynchus mykiss*) were observed with an increased expression of aromatases as compared to females [64]. Thus, evidence so far is unclear on a masculinizing or feminizing role of brain in salmonids. Of note, we did not analyze carefully excised brain tissues here but whole heads, thus the importance of gene expression in other types of tissues remains unclear. Further studies that specifically concentrate on brain tissues are necessary to confirm our first observation.

In salmonids, gonadal precursor cells typically differentiate during the weeks that follow hatching [19, 65]. During this period, the emergence of an endogenous synthesis of sexual hormones could explain why we observed a divergent response between sexes, especially if we consider that endocrine active compounds often elicit non-monotonic dose-responses [66, 67]. Such dose-effects could explain why we observed strong sex-specific responses to EE2 at hatching day and why these responses partly declined towards the juvenile stage we sampled next. Apart from the likely effects of EE2 on normal development of male and female phenotypes, exposure to EE2 also affected the expression of genes linked to many other physiological systems, including, for example, various aspects of the development of the nervous system, of skeletal muscles and of insulin metabolism. Such effects could have been responsible for the observed delay of sex differentiation. The delay does not seem to be simply stress-related, because exposure to *P. fluorescens* during embryogenesis that is known to slow down embryo development [68–70] did not cause analogous delays in sex differentiation.

## Conclusions

Exposure to high concentrations of EE2 during juvenile or early juvenile stages has been shown to induce sex reversal in many fishes. Low and ecologically relevant concentrations of EE2, i.e. concentrations that have been observed in polluted rivers, would still affect sex determination if they tipped the balance at early steps of sex differentiation so that all individuals follow the developmental process that leads to the female phenotype regardless of their sdY genotype (i.e., sex reversal of genetic males). If this were so, and if gene expression were then more influenced by gonadal development than by genetic sex, EE2 would probably not be expected to show strong sex-genotype specific effects on gene expression during later stages of sex differentiation. However, we found that exposure to EE2 during early embryogenesis leads to strong sex-genotype specific effects on gene expression after the onset of sex differentiation. Such sex-genotype specific effects suggest that low concentrations of EE2 do not tip the balance at early steps of sex differentiation. It still needs to be tested if the sex-genotype specific effects of EE2 on gene expression cause sex-specific toxic effects of EE2.

## Additional file

Additional file 1: **Figure S1.** Breeding blocks and sampling. **Figure S2.** Representative examples of the four categories of gonad categorization. **Figure S3.** EE2 concentrations in EE2-treated 200L tanks over time. **Figure S4.** Impact of DNA contamination on transcriptome analysis. **Figure S5.** Differential gene expression of control and EE2-treated males or females at different developmental stages. **Figure S6.** Frequencies of undifferentiated juveniles in control and EE2-treated groups. **Table S1.** Top 25 gene ontology terms enriching the genes of EE2-treated males at embryo stage. **Table S2.** Top 25 gene ontology terms enriching the genes of EE2-treated males at hatching. **Table S3.** Top 25 gene ontology terms enriching the genes of EE2-treated females at hatching. **Table S4.** Top 25 gene ontology terms enriching the genes of EE2-treated females at first feeding. **Table S5.** Results for classical sex-related genes. **Table S6.** Likelihood ratio test on rate of undifferentiated gonads over time and treatment. **Table S7.** RNA quality and RNA-sequencing. (DOCX 4195 kb)

## Data Availability

The gene expression data are at https://www.ncbi.nlm.nih.gov/bioproject/PRJNA388031. All other data are available from the Dryad Digital Repository: 10.5061/dryad.ms12t45.

## Abbreviations

CPM: Count per million
dpf: Days post fertilization
EE2: 17-alpha-ethinylestradiol
FDR: False discovery rate
GO: Gene ontology
T_0_: 7 days after water exchange
T_0_: Day of water exchange
