# Correction to: Sex-specific changes in gene expression in response to estrogen pollution around the onset of sex differentiation in grayling (Salmonidae)

**DOI:** 10.1186/s12864-020-06782-w

**Published:** 2020-05-20

**Authors:** Oliver M. Selmoni, Diane Maitre, Julien Roux, Laetitia G. E. Wilkins, Lucas Marques da Cunha, Etienne L. M. Vermeirssen, Susanne Knörr, Marc Robinson-Rechavi, Claus Wedekind

**Affiliations:** 1 Department of Ecology and Evolution Biophore, University of Lausanne, Lausanne, Switzerland; 2 Swiss Institute of Bioinformatics, Lausanne, Switzerland; 3 Swiss Centre for Applied Ecotoxicology Eawag-EPFL, Dübendorf, Switzerland; 4 Aquatic Ecology and Toxicology Group Center of Organismic Studies, University of Heidelberg, Heidelberg, Germany; 5 Present Address: Swiss Federal Institute of Technology (EPFL), 1015 Lausanne, Switzerland; 6 Present Address: Department of Biomedicine, University of Basel, 4031 Basel, Switzerland; 7 Present Address: Department of Environmental Sciences, Policy and Management, University of California, Berkeley, CA 94720 USA

**Correction to: BMC Genomics (2019) 20:583**

**https://doi.org/10.1186/s12864-019-5955-z**

Following publication of the original article [1], the authors reported an error in the Results section. Under the heading “Gene ontology enrichment analysis”, they wrote:

“In females at the juvenile stage, GO terms associated to glycogen metabolism (glycogen metabolic processes, insulin receptor signalling pathway) and to heart development were enriched for genes down-regulated under EE2.”

This text should be corrected as follows:

“In females at the juvenile stage, GO terms associated to glycogen metabolism (glycogen metabolic processes, insulin receptor signalling pathway) and to heart development were enriched for genes up-regulated under EE2.”

The enrichment as noted in Table S4 remains correct.

The authors wish to acknowledge Dr. Anke Lange, University of Exeter, who discovered the mistake and reported it.
