# Supplementary material for: Sex-specific changes in gene expression in response to estrogen pollution around the onset of sex differentiation in grayling (Salmonidae)
Source: BMC Genomics. 2019 Jul 15;20:583. doi: 10.1186/s12864-019-5955-z (PMC6631537; doi:10.1186/s12864-019-5955-z)
Supplement: Supplementary file 1 — Figure S1. Breeding blocks and sampling. Figure S2. Representative examples of the four categories of gonad categorization. Figure S3. EE2 concentrations in EE2-treated 200L tanks over time. Figure S4. Impact of DNA contamination on transcriptome analysis. Figure S5. Differential gene expression of control and EE2-treated males or females at different developmental stages. Figure S6. Frequencies of undifferentiated juveniles in control and EE2-treated groups. Table S1. Top 25 gene ontology terms enriching the genes of EE2-treated males at embryo stage. Table S2. Top 25 gene ontology terms enriching the genes of EE2-treated males at hatching. Table S3. Top 25 gene ontology terms enriching the genes of EE2-treated females at hatching. Table S4. Top 25 gene ontology terms enriching the genes of EE2-treated females at first feeding. Table S5. Results for classical sex-related genes. Table S6. Likelihood ratio test on rate of undifferentiated gonads over time and treatment. Table S7. RNA quality and RNA-sequencing. (DOCX 4195 kb) [file 12864_2019_5955_MOESM1_ESM.docx]

**Additional file**

**Sex-specific changes in gene expression in response to estrogen pollution around the onset of sex differentiation in grayling (Salmonidae)**

### Oliver M. Selmoni^1,5*^, Diane Maitre^1*^, Julien Roux^1,2,6^, Laetitia G. E. Wilkins^1,7^, Lucas Marques da Cunha^1^, Etienne L. M. Vermeirssen^3^, Susanne Knörr^4^, Marc Robinson-Rechavi^1,2#^, Claus Wedekind^1#^

#### ^1^ Department of Ecology and Evolution, Biophore, University of Lausanne, Lausanne, Switzerland

^2^ Swiss Institute of Bioinformatics, Lausanne, Switzerland

^3^ Swiss Centre for Applied Ecotoxicology Eawag-EPFL, Dübendorf, Switzerland

^4^ Aquatic Ecology and Toxicology Group, Center of Organismic Studies, University of Heidelberg, Heidelberg, Germany

#### * equal contributors

#### ^#^ shared senior authors

Present addresses:

^5^ Swiss Federal Institute of Technology (EPFL), 1015 Lausanne, Switzerland.

^6^ Department of Biomedicine, University of Basel, 4031 Basel, Switzerland

^7^ Department of Environmental Sciences, Policy and Management, University of California, Berkeley, CA 94720, USA

Correspondence: Claus Wedekind, [claus.wedekind@unil.ch](mailto:claus.wedekind@unil.ch), orcid.org/0000-0001-6143-4716

**Content**

*Supplementary Figures:*

Figure S1: Breeding blocks and sampling.

Figure S2: Representative examples of the four categories of gonad categorization.

Figure S3: EE2 concentrations in EE2-treated 200L tanks over time.

Figure S4: Impact of DNA contamination on transcriptome analysis.

Figure S5: Differential gene expression of control and EE2-treated males or females at different developmental stages.

Figure S6: Frequencies of undifferentiated juveniles in control and EE2-treated groups.

*Supplementary Tables:*

Table S1: Top 25 gene ontology terms enriching the genes of EE2-treated males at embryo stage.

Table S2: Top 25 gene ontology terms enriching the genes of EE2-treated males at hatching.

Table S3: Top 25 gene ontology terms enriching the genes of EE2-treated females at hatching.

Table S4: Top 25 gene ontology terms enriching the genes of EE2-treated females at first feeding.

Table S5: Results for classical sex-related genes.

Table S6: Likelihood ratio test on rate of undifferentiated gonads over time and treatment.

Table S7: RNA quality and RNA-sequencing.

**Figure S1.** Breeding blocks and sampling. Two full-factorial breeding blocks crossing 4 females (IDs: “BBE” – “BBH” and “BBK” – “BBN”) with 5 males each (IDs: “S493” – “S497” and “S504” – “S508”) resulted in 40 different sibgroups. Individuals were raised singly in 24-well plates until 40 dpf (day post fertilization; the tables give the numbers of embryos per sibgroup that were EE2-treated or controls). At 40 dpf, i.e. around the end of the yolk-sac stage, the juveniles were distributed to aquaria (indicated with the grey shadings). For transcriptomics (red arrows), EE2-treated and control samples were taken from the same five sibgroups (sharing the mother BBH) at 21, 31, and 52 dpf. To make the last sampling of these sibgroups possible, 5 juveniles per sibgroup and treatment were separated in net cages within the aquaria. Families were pooled for the remaining sampling for histological analysis (green arrows) at the time points 51, 79, 107, 135, 159-163 dpf. Individuals sampled at the last two time points were also genetically sexed.


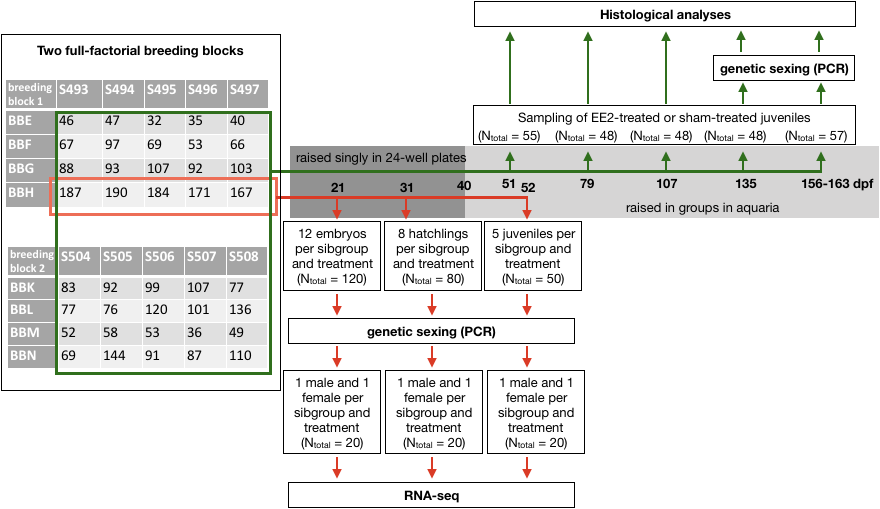


**Figure S2.** Examples of (a) undifferentiated gonads at 159-163 dpf (genetic male; arrows mark oogonia), (b) immature testis at 79 dpf (genetic sex unknown; A = spermatogonia, B = spermatocytes, C = Sertoli cells), (c) testis-to-ovary at 135 dpf (genetic female; A = perinucleolar oocytes, B = spermatogonia, C = spermatocytes), and (d) ovary at 159-163 dpf (genetic female; perinuclear oocytes). Samples were fixed in Davidsons, embedded in paraffin and HE-stain.


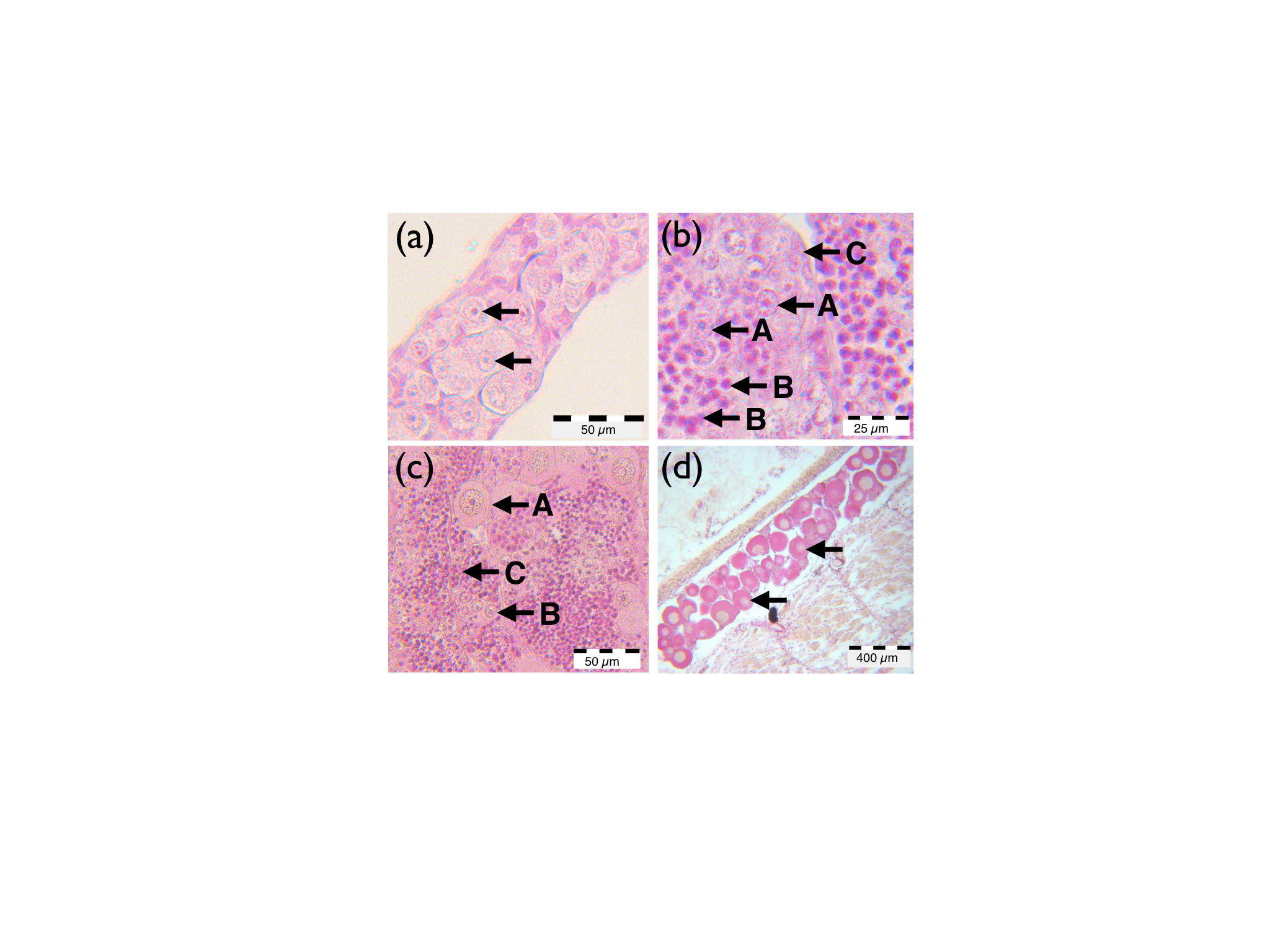


**Figure S3.** EE2 concentrations in EE2-treated 200L tanks as set at start (40 *dpf,* i.e. the day when fish were transferred into the tanks) and measured in 7-day periods, starting each 1 hour after the weekly water exchange (20% of the water replaced with fresh lake water at 1 ng/L EE2; grey boxes and symbols; T_0_) and 7 days later before the next water exchange (open boxes and symbols; T_7_). The grey and open boxes represent medians of monthly means: 4 consecutive T_0_ and 4 consecutive T_7_ samples per tank had each been pooled to reduce the number of analyses to N = 32, i.e. 4 tanks x 4 four-week intervals x 2 time points (with the last interval being a five-week interval). One unexplained outlier (5.1 ng/L) at T_0_ of the second sampling period (75-103 *dpf*) was excluded from the graph and all analyses. Its corresponding T_7_ measurement of 0.09 ng/L was very close to the ones of the other 3 tanks, i.e. the outlier is unlikely to reflect the EE2 concentration in the tank and seems instead be due to accidental contamination of some probes after sampling.


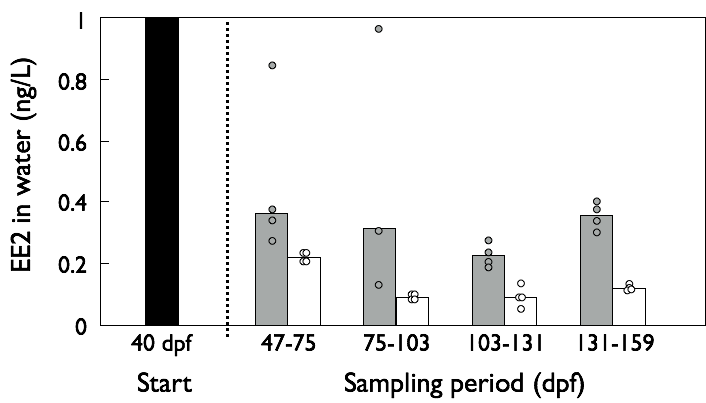


**Figure S4.** Impact of DNA contamination on transcriptome analysis.

(A) Relation between the fraction of mapped reads and the 260nm/280nm absorbance ratio prior to library preparation. (B) Principal components of gene expression from a global PCA, with transcripts coloured by the 260nm/280nm absorbance ratio prior to library preparation.

**(A)**

**
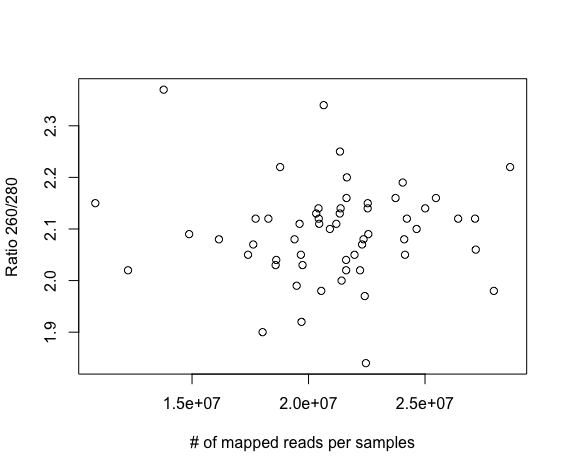
**

**(B)**


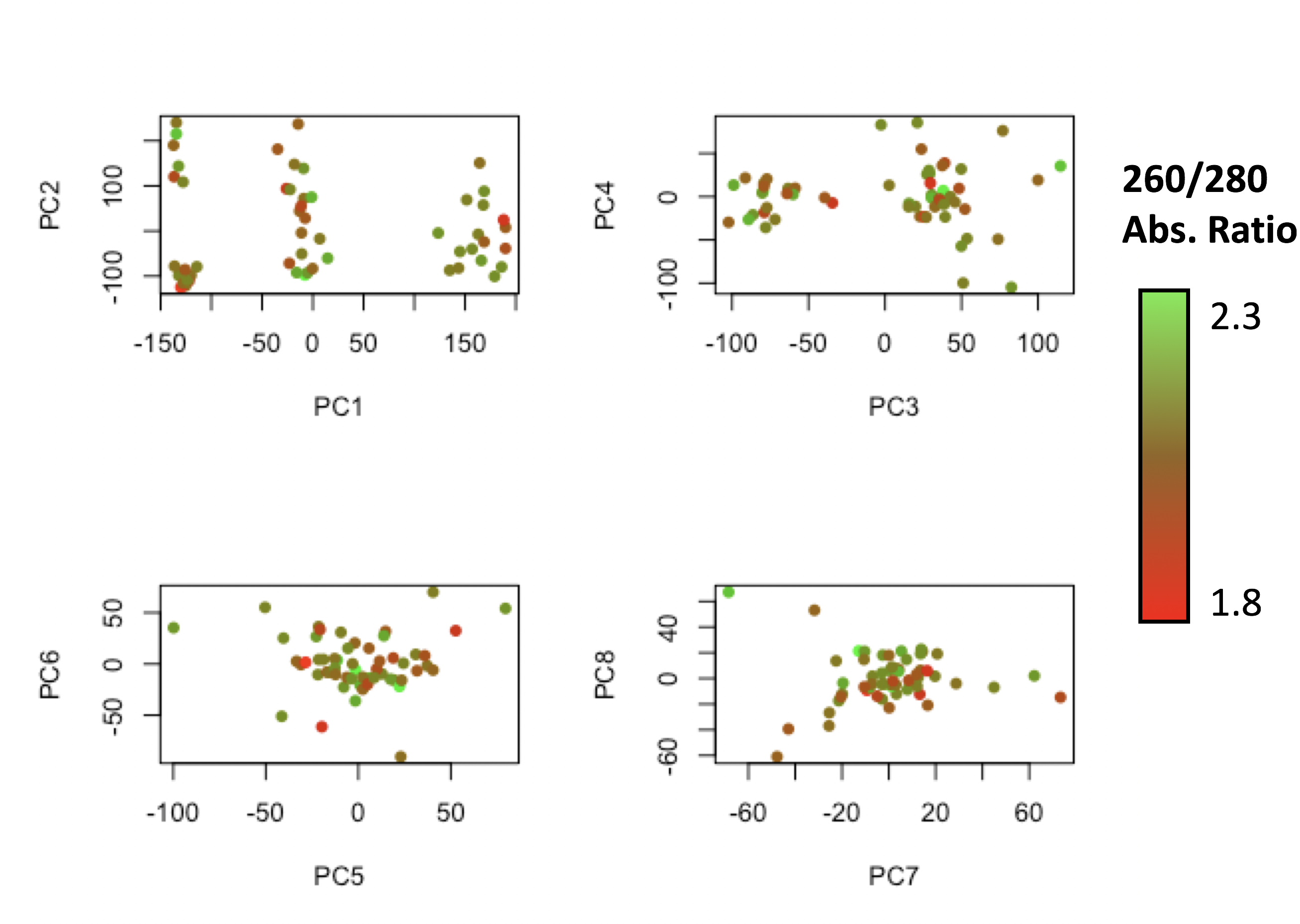


**Figure S5.** Differential gene expression of control and EE2-treated males or females at different developmental stages. The left panels show for each gene the log-fold change in expression between the two conditions of the contrast (positive: more expressed under control, negative: more expressed under EE2 treatment) and the average log-expression. Red dots indicate genes who are differentially expressed at q<0.15 (in the third figure of row c) green dots represent q<0.25). The right panels give the distribution of the p-values associated to the tests of differential expression for each gene (before q-value correction). A left skew indicates more genes found as differentially expressed than expected by chance. A flat distribution represents what we would expected if there is no difference in gene expression between the conditions. A right skew suggests a lack of accuracy (and statistical power) in the model, since fewer genes were detected as differentially expressed than expect by chance.


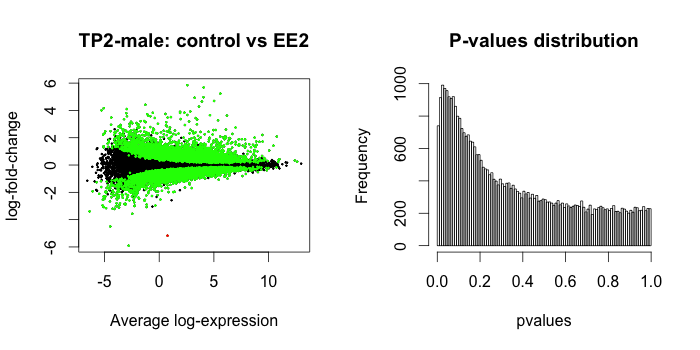

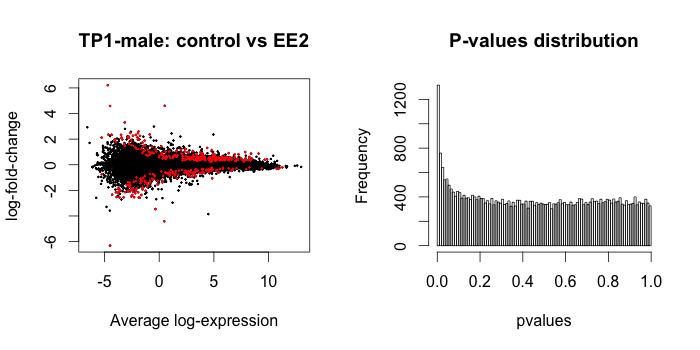


a)

Male, embryos


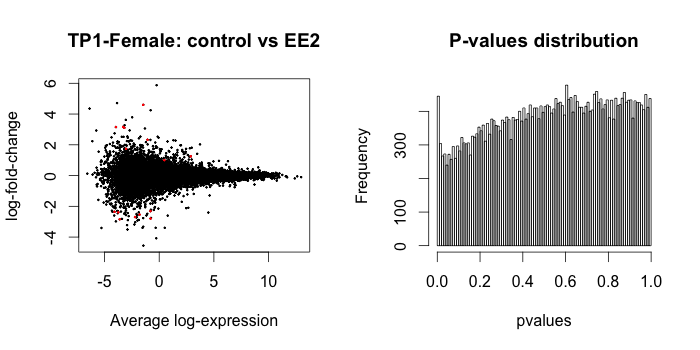


b))

Female, embryos


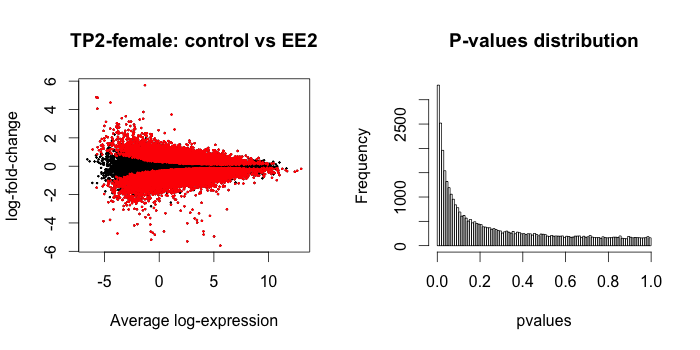

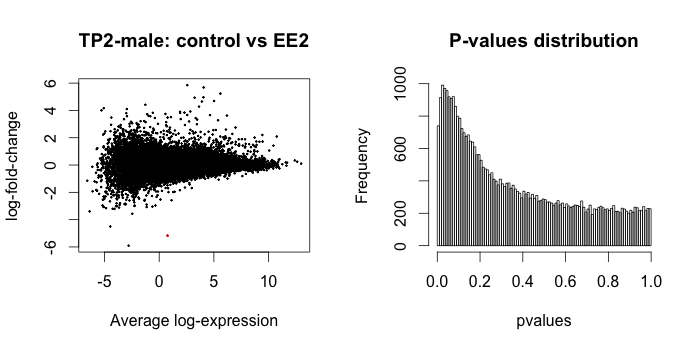


Male, hatching

c)

Female, hatching

d)


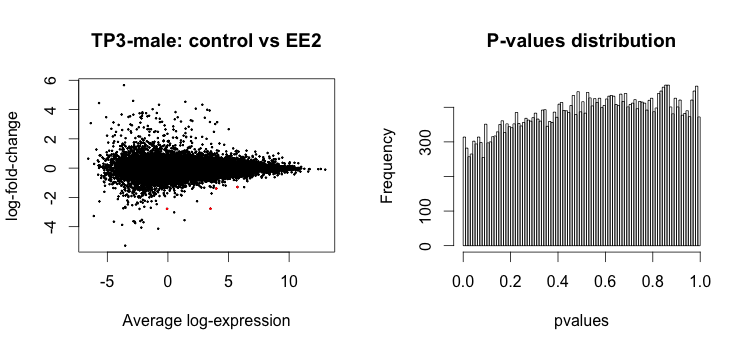


Male, juveniles

e)


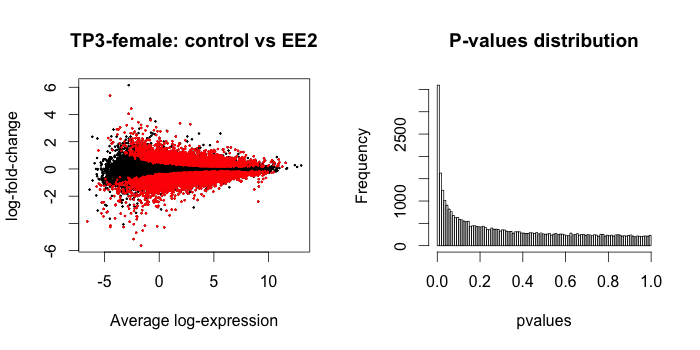


Female, juveniles

f)

**Figure S6.** Mean frequencies of undifferentiated juveniles (i.e., no testis nor ovarian tissues) when exposed to EE2 (closed symbols) or not (controls, open symbols). See Table S6 for statistics and underlying assumption. All undifferentiated juveniles at the last two sampling periods were genetic males (representing 80% of all genetic males). The figure shows the average frequencies per sex to control for (non-significant) variance in sex ratios per day and treatment. However, the overall sex ratio at the last two sampling periods was not significantly different from 50:50. Equal sex ratio was therefore assumed for first three sampling dates.
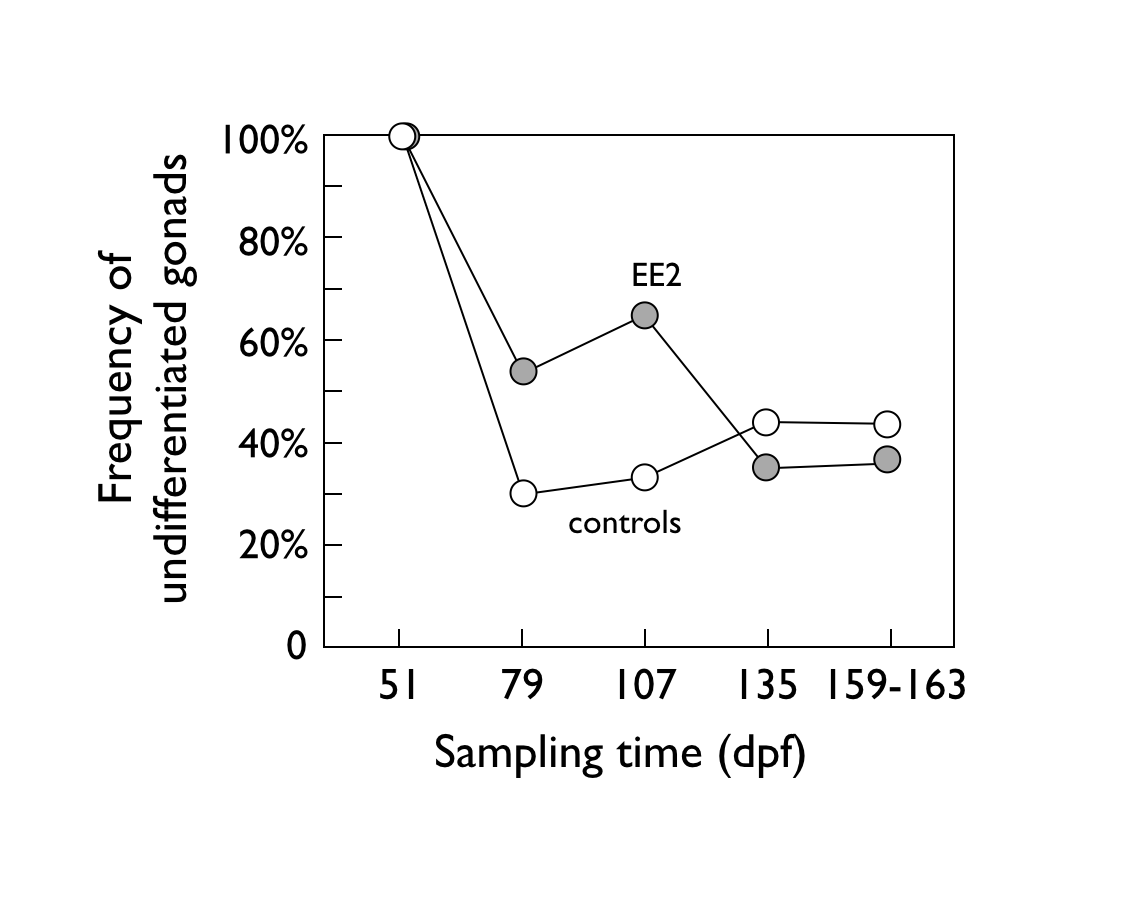


**Table S1.** Top 25 gene ontology (GO) terms enriched in the down- or up-regulated genes in EE2-treated males at embryo stage (see Fig. S3a).

|  | GO identifier | p-value | in DE | | in ALL | | Biological process |  |
| --- | --- | --- | --- | --- | --- | --- | --- | --- |
| a) Down regulated | | | | | | | |  |
|  | GO:0015276 | 2.63E-05 | 2 | | 3 | | ligand-gated ion channel activity |  |
|  | GO:0004983 | 6.66E-05 | 2 | | 4 | | neuropeptide Y receptor activity |  |
|  | GO:0004930 | 0.000616 | 8 | | 590 | | G-protein coupled receptor activity |  |
|  | GO:0007186 | 0.001849 | 8 | | 686 | | G-protein coupled receptor signaling pathway |  |
|  | GO:0015935 | 0.00294 | 2 | | 23 | | small ribosomal subunit |  |
|  | GO:0035025 | 0.003254 | 1 | | 1 | | positive regulation of Rho protein signal transduction |  |
|  | GO:0016021 | 0.003371 | 16 | | 2412 | | integral component of membrane |  |
|  | GO:0045211 | 0.004248 | 2 | | 31 | | postsynaptic membrane |  |
|  | GO:0051298 | 0.008632 | 1 | | 3 | | centrosome duplication |  |
|  | GO:0005840 | 0.008988 | 4 | | 220 | | ribosome |  |
|  | GO:0006412 | 0.010346 | 4 | | 231 | | translation |  |
|  | GO:0003735 | 0.01059 | 4 | | 231 | | structural constituent of ribosome |  |
|  | GO:0009055 | 0.015549 | 2 | | 55 | | electron carrier activity |  |
|  | GO:0004985 | 0.018343 | 1 | | 5 | | opioid receptor activity |  |
|  | GO:0042773 | 0.019024 | 1 | | 6 | | ATP synthesis coupled electron transport |  |
|  | GO:0016829 | 0.021314 | 1 | | 7 | | lyase activity |  |
|  | GO:0045028 | 0.022808 | 1 | | 7 | | G-protein coupled purinergic nucleotide receptor activity |  |
|  | GO:0019894 | 0.030612 | 1 | | 10 | | kinesin binding |  |
|  | GO:0005230 | 0.033834 | 2 | | 90 | | extracellular ligand-gated ion channel activity |  |
|  | GO:0008360 | 0.03809 | 1 | | 14 | | regulation of cell shape |  |
|  | GO:0000015 | 0.03978 | 1 | | 12 | | phosphopyruvate hydratase complex |  |
|  | GO:0004634 | 0.03978 | 1 | | 12 | | phosphopyruvate hydratase activity |  |
|  | GO:0006606 | 0.04115 | 1 | | 15 | | protein import into nucleus |  |
|  | GO:0005813 | 0.042001 | 1 | | 17 | | centrosome |  |
|  | GO:0030036 | 0.043491 | 2 | | 105 | | actin cytoskeleton organization |  |
| b) Up regulated | | | | | | | | |
|  | GO:0031492 | 5.14E-06 | | 3 | | 7 | nucleosomal DNA binding |  |
|  | GO:0007389 | 2.16E-05 | | 2 | | 2 | pattern specification process |  |
|  | GO:0033829 | 2.16E-05 | | 2 | | 2 | O-fucosylpeptide 3-beta-N-acetylglucosaminyltransferase activity |  |
|  | GO:0000785 | 3.93E-05 | | 3 | | 13 | chromatin |  |
|  | GO:0005875 | 0.000603 | | 2 | | 7 | microtubule associated complex |  |
|  | GO:0005525 | 0.0015 | | 11 | | 733 | GTP binding |  |
|  | GO:0006886 | 0.001676 | | 8 | | 434 | intracellular protein transport |  |
|  | GO:0007264 | 0.002035 | | 9 | | 547 | small GTPase mediated signal transduction |  |
|  | GO:0007186 | 0.002929 | | 10 | | 686 | G-protein coupled receptor signaling pathway |  |
|  | GO:0016757 | 0.003934 | | 3 | | 61 | transferase activity, transferring glycosyl groups |  |
|  | GO:0007596 | 0.006622 | | 2 | | 25 | blood coagulation |  |
|  | GO:0015031 | 0.007365 | | 7 | | 432 | protein transport |  |
|  | GO:0045893 | 0.008228 | | 2 | | 27 | positive regulation of transcription, DNA-templated |  |
|  | GO:0004966 | 0.010541 | | 1 | | 2 | galanin receptor activity |  |
|  | GO:0007194 | 0.010541 | | 1 | | 2 | negative regulation of adenylate cyclase activity |  |
|  | GO:0007204 | 0.010541 | | 1 | | 2 | positive regulation of cytosolic calcium ion concentration |  |
|  | GO:0045900 | 0.010649 | | 1 | | 2 | negative regulation of translational elongation |  |
|  | GO:0030173 | 0.011517 | | 2 | | 32 | integral component of Golgi membrane |  |
|  | GO:0008378 | 0.01413 | | 2 | | 35 | galactosyltransferase activity |  |
|  | GO:0000808 | 0.015286 | | 1 | | 3 | origin recognition complex |  |
|  | GO:0006184 | 0.015529 | | 6 | | 386 | GTP catabolic process |  |
|  | GO:0005664 | 0.015596 | | 1 | | 3 | nuclear origin of replication recognition complex |  |
|  | GO:0032783 | 0.019422 | | 1 | | 4 | ELL-EAF complex |  |
|  | GO:0045670 | 0.019633 | | 1 | | 4 | regulation of osteoclast differentiation |  |
| GO terms ranked according to p-value of enrichment. *In DE* = number of down- or up-regulated genes (q<0.15); *in All =* total number of genes annotated with the term. | | | | | | | |  |

**Table S2.** Top 25 GO-terms enriched in the down- or up-regulated genes in EE2-treated males at hatching (see Fig. S3c).

|  | GO identifier | p-value | in DE | | in ALL | | Biological process |  |  |
| --- | --- | --- | --- | --- | --- | --- | --- | --- | --- |
| a) Down regulated | | | | | | | | |  |
|  | GO:0004175 | 1.13E-17 | 29 | | 42 | | endopeptidase activity |  |  |
|  | GO:0004298 | 3.64E-16 | 29 | | 46 | | threonine-type endopeptidase activity |  |  |
|  | GO:0005839 | 3.64E-16 | 29 | | 46 | | proteasome core complex |  |  |
|  | GO:0051603 | 3.64E-16 | 29 | | 46 | | proteolysis involved in cellular protein catabolic process |  |  |
|  | GO:0019773 | 1.23E-08 | 13 | | 19 | | proteasome core complex, alpha-subunit complex |  |  |
|  | GO:0004129 | 8.98E-05 | 15 | | 41 | | cytochrome-c oxidase activity |  |  |
|  | GO:0006281 | 0.000153 | 22 | | 87 | | DNA repair |  |  |
|  | GO:0006012 | 0.000165 | 5 | | 6 | | galactose metabolic process |  |  |
|  | GO:0003747 | 0.000244 | 4 | | 4 | | translation release factor activity |  |  |
|  | GO:0000077 | 0.000276 | 5 | | 7 | | DNA damage checkpoint |  |  |
|  | GO:0006412 | 0.000287 | 48 | | 231 | | translation |  |  |
|  | GO:0006165 | 0.00036 | 7 | | 13 | | nucleoside diphosphate phosphorylation |  |  |
|  | GO:0006183 | 0.00036 | 7 | | 13 | | GTP biosynthetic process |  |  |
|  | GO:0006228 | 0.00036 | 7 | | 13 | | UTP biosynthetic process |  |  |
|  | GO:0006241 | 0.00036 | 7 | | 13 | | CTP biosynthetic process |  |  |
|  | GO:0006122 | 0.000611 | 5 | | 7 | | mitochondrial electron transport, ubiquinol to cytochrome c |  |  |
|  | GO:0004550 | 0.000658 | 7 | | 14 | | nucleoside diphosphate kinase activity |  |  |
|  | GO:0006269 | 0.000762 | 4 | | 5 | | DNA replication, synthesis of RNA primer |  |  |
|  | GO:0005849 | 0.001085 | 4 | | 5 | | mRNA cleavage factor complex |  |  |
|  | GO:0051297 | 0.001189 | 5 | | 9 | | centrosome organization |  |  |
|  | GO:0006511 | 0.001329 | 31 | | 166 | | ubiquitin-dependent protein catabolic process |  |  |
|  | GO:0006487 | 0.00157 | 7 | | 16 | | protein N-linked glycosylation |  |  |
|  | GO:0030144 | 0.00168 | 6 | | 12 | | alpha-1,6-mannosylglycoprotein 6-beta-N-acetylglucosaminyltransferase activity |  |  |
|  | GO:0006367 | 0.001731 | 6 | | 12 | | transcription initiation from RNA polymerase II promoter |  |  |
|  | GO:0003735 | 0.00191 | 45 | | 231 | | structural constituent of ribosome |  |  |
| b) Up regulated | | | | | | | | | |
|  | GO:0003774 | 9.56E-10 | | 45 | | 160 | motor activity |  |  |
|  | GO:0016459 | 1.43E-09 | | 44 | | 156 | myosin complex |  |  |
|  | GO:0005882 | 4.52E-09 | | 29 | | 88 | intermediate filament |  |  |
|  | GO:0005198 | 5.23E-08 | | 49 | | 219 | structural molecule activity |  |  |
|  | GO:0031105 | 2.46E-05 | | 9 | | 18 | septin complex |  |  |
|  | GO:0045095 | 2.62E-05 | | 7 | | 11 | keratin filament |  |  |
|  | GO:0005856 | 3.15E-05 | | 42 | | 201 | cytoskeleton |  |  |
|  | GO:0042157 | 9.38E-05 | | 8 | | 17 | lipoprotein metabolic process |  |  |
|  | GO:0003730 | 0.000102 | | 4 | | 4 | mRNA 3'-UTR binding |  |  |
|  | GO:0005201 | 0.00013 | | 15 | | 47 | extracellular matrix structural constituent |  |  |
|  | GO:0006096 | 0.000155 | | 16 | | 58 | glycolytic process |  |  |
|  | GO:0005581 | 0.00029 | | 12 | | 35 | collagen trimer |  |  |
|  | GO:0019904 | 0.000428 | | 11 | | 36 | protein domain specific binding |  |  |
|  | GO:0031032 | 0.000452 | | 5 | | 8 | actomyosin structure organization |  |  |
|  | GO:0019001 | 0.000578 | | 14 | | 55 | guanyl nucleotide binding |  |  |
|  | GO:0031683 | 0.000578 | | 14 | | 55 | G-protein beta/gamma-subunit complex binding |  |  |
|  | GO:0006869 | 0.000721 | | 10 | | 29 | lipid transport |  |  |
|  | GO:0016012 | 0.000779 | | 6 | | 13 | sarcoglycan complex |  |  |
|  | GO:0007049 | 0.000966 | | 19 | | 90 | cell cycle |  |  |
|  | GO:0030054 | 0.000987 | | 6 | | 11 | cell junction |  |  |
|  | GO:0005219 | 0.000993 | | 6 | | 11 | ryanodine-sensitive calcium-release channel activity |  |  |
|  | GO:0006874 | 0.000993 | | 6 | | 11 | cellular calcium ion homeostasis |  |  |
|  | GO:0005000 | 0.001273 | | 4 | | 6 | vasopressin receptor activity |  |  |
|  | GO:0030335 | 0.001625 | | 4 | | 6 | positive regulation of cell migration |  |  |
|  | GO:0043022 | 0.002216 | | 4 | | 7 | ribosome binding |  |  |
| GO terms ranked according to p-value. *In DE* = number of down- or up-regulated genes (q<0.15); *in All =* total number of genes annotated with the term. | | | | | | | |  |  |

**Table S3.** Top 25 GO-terms enriched in the down- or up-regulated genes in EE2-treated females at hatching (see Fig. S3d).

|  | GO identifier | p-value | in DE | | in ALL | | Biological process |  |  |
| --- | --- | --- | --- | --- | --- | --- | --- | --- | --- |
| a) Down regulated | | | | | | | | |  |
|  | GO:0005515 | 4.11E-13 | 2498 | | 7275 | | protein binding |  |  |
|  | GO:0007155 | 5.39E-13 | 221 | | 447 | | cell adhesion |  |  |
|  | GO:0006468 | 1.79E-10 | 439 | | 1120 | | protein phosphorylation |  |  |
|  | GO:0016772 | 2.80E-10 | 459 | | 1174 | | transferase activity, transferring phosphorus-containing groups |  |  |
|  | GO:0004672 | 6.32E-10 | 416 | | 1054 | | protein kinase activity |  |  |
|  | GO:0005524 | 1.72E-09 | 800 | | 2152 | | ATP binding |  |  |
|  | GO:0005488 | 7.53E-09 | 253 | | 544 | | binding |  |  |
|  | GO:0005024 | 3.11E-08 | 17 | | 19 | | transforming growth factor beta-activated receptor activity |  |  |
|  | GO:0004674 | 3.85E-07 | 240 | | 595 | | protein serine/threonine kinase activity |  |  |
|  | GO:0004675 | 6.92E-07 | 17 | | 21 | | transmembrane receptor protein serine/threonine kinase activity |  |  |
|  | GO:0004702 | 1.47E-06 | 24 | | 36 | | receptor signaling protein serine/threonine kinase activity |  |  |
|  | GO:0007178 | 1.47E-06 | 24 | | 36 | | transmembrane receptor protein serine/threonine kinase signaling pathway |  |  |
|  | GO:0007156 | 2.91E-06 | 114 | | 238 | | homophilic cell adhesion via plasma membrane adhesion molecules |  |  |
|  | GO:0006355 | 3.26E-06 | 534 | | 1637 | | regulation of transcription, DNA-templated |  |  |
|  | GO:0010468 | 3.31E-06 | 13 | | 14 | | regulation of gene expression |  |  |
|  | GO:0008017 | 6.52E-06 | 86 | | 166 | | microtubule binding |  |  |
|  | GO:0019904 | 1.45E-05 | 21 | | 36 | | protein domain specific binding |  |  |
|  | GO:0000159 | 2.76E-05 | 20 | | 35 | | protein phosphatase type 2A complex |  |  |
|  | GO:0008601 | 2.76E-05 | 20 | | 35 | | protein phosphatase type 2A regulator activity |  |  |
|  | GO:0008093 | 6.60E-05 | 17 | | 27 | | cytoskeletal adaptor activity |  |  |
|  | GO:0017124 | 6.60E-05 | 17 | | 27 | | SH3 domain binding |  |  |
|  | GO:0046847 | 6.60E-05 | 17 | | 27 | | filopodium assembly |  |  |
|  | GO:0007169 | 7.93E-05 | 42 | | 79 | | transmembrane receptor protein tyrosine kinase signaling pathway |  |  |
|  | GO:0044267 | 8.62E-05 | 17 | | 25 | | cellular protein metabolic process |  |  |
|  | GO:0005003 | 0.000119 | 21 | | 34 | | ephrin receptor activity |  |  |
| b) Up regulated | | | | | | | | | |
|  | GO:0004129 | 3.27E-09 | | 29 | | 41 | cytochrome-c oxidase activity |  |  |
|  | GO:0003735 | 2.07E-08 | | 98 | | 231 | structural constituent of ribosome |  |  |
|  | GO:0005840 | 2.62E-08 | | 94 | | 220 | ribosome |  |  |
|  | GO:0006412 | 3.60E-08 | | 97 | | 231 | translation |  |  |
|  | GO:0005739 | 6.67E-08 | | 30 | | 48 | mitochondrion |  |  |
|  | GO:0001522 | 4.52E-06 | | 14 | | 18 | pseudouridine synthesis |  |  |
|  | GO:0003899 | 2.28E-05 | | 25 | | 49 | DNA-directed RNA polymerase activity |  |  |
|  | GO:0006413 | 2.32E-05 | | 21 | | 36 | translational initiation |  |  |
|  | GO:0016272 | 5.81E-05 | | 11 | | 14 | prefoldin complex |  |  |
|  | GO:0005747 | 6.12E-05 | | 7 | | 7 | mitochondrial respiratory chain complex I |  |  |
|  | GO:0003676 | 6.58E-05 | | 504 | | 1861 | nucleic acid binding |  |  |
|  | GO:0016881 | 7.00E-05 | | 42 | | 95 | acid-amino acid ligase activity |  |  |
|  | GO:0004497 | 8.25E-05 | | 18 | | 31 | monooxygenase activity |  |  |
|  | GO:0016592 | 8.39E-05 | | 19 | | 37 | mediator complex |  |  |
|  | GO:0006122 | 8.95E-05 | | 7 | | 7 | mitochondrial electron transport, ubiquinol to cytochrome c |  |  |
|  | GO:0042981 | 0.000106 | | 36 | | 82 | regulation of apoptotic process |  |  |
|  | GO:0003743 | 0.000112 | | 27 | | 55 | translation initiation factor activity |  |  |
|  | GO:0001104 | 0.000129 | | 18 | | 35 | RNA polymerase II transcription cofactor activity |  |  |
|  | GO:0009982 | 0.000131 | | 10 | | 13 | pseudouridine synthase activity |  |  |
|  | GO:0016491 | 0.000132 | | 130 | | 402 | oxidoreductase activity |  |  |
|  | GO:0005212 | 0.000157 | | 8 | | 9 | structural constituent of eye lens |  |  |
|  | GO:0016787 | 0.000169 | | 86 | | 252 | hydrolase activity |  |  |
|  | GO:0006351 | 0.000296 | | 53 | | 148 | transcription, DNA-templated |  |  |
|  | GO:0030286 | 0.000308 | | 9 | | 16 | dynein complex |  |  |
|  | GO:0006357 | 0.00034 | | 30 | | 75 | regulation of transcription from RNA polymerase II promoter |  |  |
| GO terms ranked according to p-value. *In DE* = number of number of down- or up-regulated genes (q<0.15); *in All =* total number of genes annotated with the term. | | | | | | | |  |  |

**Table S4.** Top 25 GO-terms enriched in the down- or up-regulated genes in EE2-treated females at first feeding (see Fig. S3f).

|  | GO identifier | p-value | in DE | | in ALL | | Biological process |  |  |
| --- | --- | --- | --- | --- | --- | --- | --- | --- | --- |
| a) Down regulated | | | | | | | | |  |
|  | GO:0006412 | 9.44E-24 | 106 | | 231 | | translation |  |  |
|  | GO:0005840 | 1.08E-23 | 103 | | 220 | | ribosome |  |  |
|  | GO:0003735 | 1.69E-23 | 106 | | 231 | | structural constituent of ribosome |  |  |
|  | GO:0004129 | 1.21E-15 | 31 | | 41 | | cytochrome-c oxidase activity |  |  |
|  | GO:0015986 | 1.63E-11 | 20 | | 26 | | ATP synthesis coupled proton transport |  |  |
|  | GO:0005739 | 5.97E-10 | 27 | | 48 | | mitochondrion |  |  |
|  | GO:0005622 | 1.29E-09 | 262 | | 1266 | | intracellular |  |  |
|  | GO:0004175 | 8.80E-08 | 22 | | 42 | | endopeptidase activity |  |  |
|  | GO:0004298 | 1.09E-07 | 23 | | 46 | | threonine-type endopeptidase activity |  |  |
|  | GO:0005839 | 1.09E-07 | 23 | | 46 | | proteasome core complex |  |  |
|  | GO:0051603 | 1.09E-07 | 23 | | 46 | | proteolysis involved in cellular protein catabolic process |  |  |
|  | GO:0005743 | 2.28E-07 | 20 | | 37 | | mitochondrial inner membrane |  |  |
|  | GO:0016787 | 2.68E-07 | 68 | | 252 | | hydrolase activity |  |  |
|  | GO:0016491 | 3.02E-07 | 98 | | 402 | | oxidoreductase activity |  |  |
|  | GO:0008137 | 4.04E-07 | 14 | | 21 | | NADH dehydrogenase (ubiquinone) activity |  |  |
|  | GO:0015078 | 4.26E-07 | 19 | | 38 | | hydrogen ion transmembrane transporter activity |  |  |
|  | GO:0005737 | 8.85E-07 | 116 | | 508 | | cytoplasm |  |  |
|  | GO:0007017 | 9.94E-07 | 25 | | 57 | | microtubule-based process |  |  |
|  | GO:0003743 | 1.23E-06 | 24 | | 55 | | translation initiation factor activity |  |  |
|  | GO:0006413 | 2.36E-06 | 18 | | 36 | | translational initiation |  |  |
|  | GO:0005747 | 3.12E-06 | 7 | | 7 | | mitochondrial respiratory chain complex I |  |  |
|  | GO:0005885 | 3.99E-06 | 7 | | 7 | | Arp2/3 protein complex |  |  |
|  | GO:0034314 | 5.72E-06 | 8 | | 9 | | Arp2/3 complex-mediated actin nucleation |  |  |
|  | GO:0006457 | 6.82E-06 | 42 | | 139 | | protein folding |  |  |
|  | GO:0006122 | 7.38E-06 | 7 | | 7 | | mitochondrial electron transport, ubiquinol to cytochrome c |  |  |
| b) Up regulated | | | | | | | | | |
|  | GO:0003964 | 6.87E-15 | | 17 | | 24 | RNA-directed DNA polymerase activity |  |  |
|  | GO:0006278 | 6.87E-15 | | 17 | | 24 | RNA-dependent DNA replication |  |  |
|  | GO:0004803 | 1.03E-13 | | 22 | | 42 | transposase activity |  |  |
|  | GO:0006313 | 1.03E-13 | | 22 | | 42 | transposition, DNA-mediated |  |  |
|  | GO:0015074 | 5.03E-12 | | 22 | | 47 | DNA integration |  |  |
|  | GO:0005488 | 1.33E-07 | | 135 | | 544 | binding |  |  |
|  | GO:0008286 | 4.83E-07 | | 8 | | 11 | insulin receptor signaling pathway |  |  |
|  | GO:0003723 | 7.30E-06 | | 73 | | 411 | RNA binding |  |  |
|  | GO:0016772 | 3.28E-05 | | 191 | | 1174 | transferase activity, transferring phosphorus-containing groups |  |  |
|  | GO:0003676 | 5.76E-05 | | 260 | | 1861 | nucleic acid binding |  |  |
|  | GO:0006468 | 6.97E-05 | | 179 | | 1120 | protein phosphorylation |  |  |
|  | GO:0004672 | 7.01E-05 | | 172 | | 1054 | protein kinase activity |  |  |
|  | GO:0000123 | 7.05E-05 | | 9 | | 13 | histone acetyltransferase complex |  |  |
|  | GO:0036459 | 0.000272 | | 28 | | 98 | ubiquitinyl hydrolase activity |  |  |
|  | GO:0004842 | 0.000371 | | 40 | | 167 | ubiquitin-protein transferase activity |  |  |
|  | GO:0007507 | 0.000388 | | 4 | | 5 | heart development |  |  |
|  | GO:0004386 | 0.000429 | | 45 | | 159 | helicase activity |  |  |
|  | GO:0007169 | 0.000503 | | 22 | | 79 | transmembrane receptor protein tyrosine kinase signaling pathway |  |  |
|  | GO:0032793 | 0.000544 | | 6 | | 12 | positive regulation of CREB transcription factor activity |  |  |
|  | GO:0005524 | 0.000607 | | 332 | | 2152 | ATP binding |  |  |
|  | GO:0005007 | 0.000925 | | 6 | | 10 | fibroblast growth factor-activated receptor activity |  |  |
|  | GO:0008270 | 0.001337 | | 248 | | 1734 | zinc ion binding |  |  |
|  | GO:0003677 | 0.001341 | | 199 | | 1549 | DNA binding |  |  |
|  | GO:0005977 | 0.001571 | | 5 | | 11 | glycogen metabolic process |  |  |
|  | GO:0004674 | 0.001686 | | 99 | | 595 | protein serine/threonine kinase activity |  |  |
| GO terms ranked according to p-value. *In DE* = number of down- or up-regulated genes (q<0.15); *in All =* total number of genes annotated with the term. | | | | | | | |  |  |

**Table S5.** Results for known sex related genes.

Results are presented per gene from the reference transcriptome. For each gene, its annotation is shown, followed by the q-value corresponding to the test of differential expression between control and EE2 treated for each developmental stage, then the log fold-change (logFC) for the same tests; positive logFC = more expressed in control; negative logFC = more expressed in EE2-treatment.

|  |  | q-value |  |  |  |  |  | logFC |  |  |  |  |  |
| --- | --- | --- | --- | --- | --- | --- | --- | --- | --- | --- | --- | --- | --- |
|  |  | Embryo |  | Hatching |  | Juveniles |  | Embryo | | Hatching | | Juveniles | |
| **GeneID** | **Gene Annotation** | **Male** | **Female** | **Male** | **Female** | **Male** | **Female** | **Male** | **Female** | **Male** | **Female** | **Male** | **Female** |
| 323 | cyp1a1: Cytochrome P450 1A1 (Oncorhynchus mykiss) | 0.75600 | 0.99998 | 0.38850 | 0.08080 | 0.00000 | 0.00000 | 0.30 | -0.09 | -0.25 | 0.60 | -2.77 | -2.28 |
| 649 | CYP2D15: Cytochrome P450 2D15 (Canis familiaris) | 0.89921 | 0.99998 | 0.29814 | 0.06013 | 0.99958 | 0.01937 | 0.11 | 0.25 | -0.47 | 0.84 | 0.25 | -1.33 |
| 1628 | CYP26C1: Cytochrome P450 26C1 (Homo sapiens) | 0.79200 | 0.99998 | 0.53406 | 0.11542 | 0.99958 | 0.28764 | -0.13 | 0.17 | -0.03 | 0.29 | -0.20 | -0.25 |
| 4598 | CYP1B1: Cytochrome P450 1B1 (Homo sapiens) | 0.85232 | 0.99998 | 0.31044 | 0.34942 | 0.99958 | 0.34467 | -0.27 | 0.00 | 0.80 | -0.19 | 0.37 | 0.50 |
| 6363 | Cyp2u1: Cytochrome P450 2U1 (Mus musculus) | 0.62464 | 0.99998 | 0.45923 | 0.08697 | 0.99958 | 0.54165 | 0.51 | 0.11 | -0.22 | 0.78 | -0.91 | 0.03 |
| 6620 | CYP27C1: Cytochrome P450 27C1 (Homo sapiens) | 0.77489 | 0.99998 | 0.41823 | 0.14971 | 0.99958 | 0.28551 | 0.22 | -0.12 | -0.18 | 0.36 | -0.09 | 0.35 |
| 9041 | Cyp20a1: Cytochrome P450 20A1 (Rattus norvegicus) | 0.90659 | 0.99998 | 0.23652 | 0.12111 | 0.99958 | 0.19981 | 0.02 | 0.12 | -0.33 | 0.21 | 0.31 | -0.26 |
| 10363 | Cyp2u1: Cytochrome P450 2U1 (Mus musculus) | 0.38364 | 0.99998 | 0.47193 | 0.07750 | 0.99958 | 0.39429 | 0.69 | -0.15 | -0.17 | 0.72 | -0.73 | -0.25 |
| 10807 | Cyp4b1: Cytochrome P450 4B1 (Rattus norvegicus) | 0.83621 | 0.99998 | 0.23989 | 0.04740 | 0.99958 | 0.01602 | 0.12 | 0.08 | -0.38 | 0.56 | -0.04 | -0.85 |
| 15065 | cyp2k1: Cytochrome P450 2K1 (Oncorhynchus mykiss) | 0.90227 | 0.99998 | 0.34489 | 0.07393 | 0.99958 | 0.49764 | 0.06 | 0.32 | -0.26 | 0.54 | -0.21 | 0.09 |
| 15512 | cyp2k1: Cytochrome P450 2K1 (Oncorhynchus mykiss) | 0.86904 | 0.99998 | 0.55838 | 0.10935 | 0.99958 | 0.29713 | 0.14 | -0.11 | -0.02 | 0.50 | -0.52 | 0.30 |
| 15714 | Cyp4b1: Cytochrome P450 4B1 (Rattus norvegicus) | 0.72334 | 0.99998 | 0.32659 | 0.13527 | 0.99958 | 0.09435 | 0.46 | 0.57 | -0.64 | 0.78 | 0.51 | -1.44 |
| 16344 | Cyp2f2: Cytochrome P450 2F2 (Mus musculus) | 0.86064 | 0.99998 | 0.35861 | 0.32870 | 0.99958 | 0.43662 | -0.23 | -0.08 | -0.76 | -0.34 | 0.44 | -0.49 |
| 16634 | cyp26a1: Cytochrome P450 26A1 (Danio rerio) | 0.57357 | 0.99998 | 0.26766 | 0.05130 | 0.99958 | 0.26790 | -1.72 | 2.52 | -1.99 | 3.39 | 0.27 | -2.48 |
| 17189 | CYP2J2: Cytochrome P450 2J2 (Homo sapiens) | 0.17284 | 0.99998 | 0.43721 | 0.14678 | 0.99958 | 0.02528 | -0.76 | 0.41 | -0.31 | 0.68 | -0.29 | -1.72 |
| 17299 | cyp26b1: Cytochrome P450 26B1 (Danio rerio) | 0.91739 | 0.99998 | 0.33319 | 0.10023 | 0.99958 | 0.06890 | 0.01 | -0.66 | 0.36 | -0.60 | -0.27 | 0.96 |
| 17533 | CYP2F3: Cytochrome P450 2F3 (Capra hircus) | 0.90119 | 0.99998 | 0.29056 | 0.10924 | 0.99958 | 0.51501 | -0.11 | -0.04 | -1.42 | -2.04 | 0.47 | -0.22 |
| 18934 | CYP2J2: Cytochrome P450 2J2 (Homo sapiens) | 0.84143 | 0.99998 | 0.23652 | 0.29451 | 0.99958 | 0.52383 | 0.08 | -0.03 | -0.33 | 0.08 | -0.44 | 0.02 |
| 22590 | cyp26a1: Cytochrome P450 26A1 (Danio rerio) | 0.90273 | 0.99998 | 0.33822 | 0.04604 | 0.99958 | 0.38866 | -0.08 | 0.26 | -0.40 | 1.27 | 0.78 | -0.51 |
| 27013 | cyp2m1: Cytochrome P450 2M1 (Oncorhynchus mykiss) | 0.79827 | 0.99998 | 0.55933 | 0.16399 | 0.99958 | 0.29613 | -0.21 | -0.22 | -0.01 | 0.34 | -0.36 | -0.37 |
| 27015 | cyp2m1: Cytochrome P450 2M1 (Oncorhynchus mykiss) | 0.68438 | 0.99998 | 0.48739 | 0.06911 | 0.99958 | 0.39520 | 0.16 | 0.07 | -0.06 | 0.33 | 0.26 | 0.11 |
| 28094 | cyp2k1: Cytochrome P450 2K1 (Oncorhynchus mykiss) | 0.46037 | 0.99998 | 0.46547 | 0.39066 | 0.99958 | 0.28764 | 0.55 | 0.22 | 0.36 | -0.12 | 1.20 | -1.16 |
| 28614 | cyp26b1: Cytochrome P450 26B1 (Danio rerio) | 0.87942 | 0.99998 | 0.25339 | 0.05476 | 0.99958 | 0.22898 | 0.17 | 0.37 | -0.57 | 0.83 | -0.02 | -0.54 |
| 29241 | Cyp2j6: Cytochrome P450 2J6 (Mus musculus) | 0.61925 | 0.99998 | 0.23913 | 0.10949 | 0.99958 | 0.45475 | 0.20 | -0.06 | 0.29 | 0.25 | -0.24 | -0.08 |
| 29524 | cyp2k1: Cytochrome P450 2K1 (Oncorhynchus mykiss) | 0.50676 | 0.99998 | 0.23652 | 0.04604 | 0.99958 | 0.16279 | 0.54 | 0.12 | -0.95 | 1.25 | -0.24 | -0.67 |
| 29929 | CYP11B: Cytochrome P450 11B%2C mitochondrial (Lithobates catesbeiana) | 0.86285 | 0.99998 | 0.33431 | 0.04870 | 0.99958 | 0.26800 | -0.29 | 0.24 | -0.66 | 1.59 | 0.30 | -0.74 |
| 32481 | cyp1a1: Cytochrome P450 1A1 (Oncorhynchus mykiss) | 0.91730 | 0.99998 | 0.45175 | 0.06250 | 0.00009 | 0.00003 | 0.04 | 0.28 | -0.34 | 1.53 | -2.79 | -2.71 |
| 34261 | Cyp4f6: Cytochrome P450 4F6 (Rattus norvegicus) | 0.90744 | 0.99998 | 0.37099 | 0.33838 | 0.99958 | 0.34598 | 0.05 | -0.11 | 0.35 | -0.16 | 0.03 | -0.34 |
| 34330 | cyp3a40: Cytochrome P450 3A40 (Oryzias latipes) | 0.90153 | 0.99998 | 0.39431 | 0.04997 | 0.99958 | 0.24600 | 0.08 | -0.10 | 0.27 | 0.91 | -0.80 | -0.49 |
| 36342 | cyp1a1: Cytochrome P450 1A1 (Stenotomus chrysops) | 0.88273 | 0.75901 | 0.25186 | 0.26280 | 0.99958 | 0.38459 | 0.33 | 1.94 | 1.36 | -0.62 | -0.69 | -0.50 |
| 37086 | CYP1B1: Cytochrome P450 1B1 (Homo sapiens) | 0.87939 | 0.99998 | 0.28390 | 0.27007 | 0.99958 | 0.36158 | -0.13 | -0.10 | 0.45 | -0.23 | -1.07 | 0.32 |
| 37087 | CYP1B1: Cytochrome P450 1B1 (Homo sapiens) | 0.75778 | 0.99998 | 0.23652 | 0.28576 | 0.99958 | 0.23698 | 0.25 | -0.40 | 0.59 | 0.18 | -0.89 | -0.46 |
| 41532 | cyp2m1: Cytochrome P450 2M1 (Oncorhynchus mykiss) | 0.90558 | 0.99998 | 0.49948 | 0.22517 | 0.99958 | 0.54920 | -0.06 | -0.27 | 0.13 | 0.34 | -0.22 | -0.01 |
| 41627 | CYP4V2: Cytochrome P450 4V2 (Pongo abelii) | 0.80919 | 0.99998 | 0.33851 | 0.04631 | 0.99958 | 0.04314 | -0.46 | 0.19 | 0.82 | 2.33 | 1.39 | -2.44 |
| 42497 | cyp2k1: Cytochrome P450 2K1 (Oncorhynchus mykiss) | 0.89495 | 0.99998 | 0.50165 | 0.31273 | 0.99958 | 0.07238 | -0.07 | -0.21 | -0.09 | 0.13 | -0.45 | 0.69 |
| 43234 | CYP27C1: Cytochrome P450 27C1 (Homo sapiens) | 0.84171 | 0.99998 | 0.25616 | 0.40383 | 0.99958 | 0.03737 | -0.16 | -0.17 | 0.52 | 0.03 | 0.27 | 1.37 |
| 43588 | cyp26a1: Cytochrome P450 26A1 (Danio rerio) | 0.74555 | 0.99998 | 0.34682 | 0.16688 | 0.99958 | 0.35207 | -0.14 | -0.08 | 0.18 | -0.23 | 0.21 | -0.17 |
| 44109 | Cyp1b1: Cytochrome P450 1B1 (Mus musculus) | 0.42029 | 0.99998 | 0.41770 | 0.39913 | 0.99958 | 0.07106 | 0.76 | -0.33 | 0.34 | 0.06 | -0.76 | -1.14 |
| 45274 | cyp2k1: Cytochrome P450 2K1 (Oncorhynchus mykiss) | 0.90273 | 0.99998 | 0.23652 | 0.25932 | 0.99958 | 0.36650 | 0.15 | -0.50 | -0.98 | 0.35 | -1.07 | -0.50 |
| 47697 | CYP2J2: Cytochrome P450 2J2 (Homo sapiens) | 0.91517 | 0.99998 | 0.49629 | 0.07445 | 0.99958 | 0.11241 | -0.02 | -0.02 | 0.10 | 0.60 | -0.03 | -0.50 |
| 48343 | CYP1B1: Cytochrome P450 1B1 (Homo sapiens) | 0.57206 | 0.99998 | 0.26863 | 0.40184 | 0.99958 | 0.07744 | 0.41 | -0.47 | 0.44 | 0.03 | -1.17 | 0.90 |
| 48344 | CYP1B1: Cytochrome P450 1B1 (Homo sapiens) | 0.89172 | 0.99998 | 0.53780 | 0.39094 | 0.99958 | 0.16433 | 0.15 | -0.47 | -0.06 | -0.06 | -1.83 | 1.01 |
| 11619 | Ar: Androgen receptor (Rattus norvegicus) | 0.89752 | 0.99998 | 0.25035 | 0.15091 | 0.99958 | 0.28488 | 0.07 | -0.12 | -0.38 | 0.30 | -0.31 | 0.25 |
| 21581 | Ar: Androgen receptor (Rattus norvegicus) | 0.87477 | 0.99998 | 0.48419 | 0.28047 | 0.99958 | 0.11128 | -0.10 | -0.01 | 0.09 | -0.15 | -0.15 | 0.44 |
| 24462 | AR: Androgen receptor (Crocuta crocuta) | 0.60612 | 0.99998 | 0.55300 | 0.34920 | 0.99958 | 0.46471 | 0.49 | -0.57 | -0.04 | -0.18 | -0.23 | -0.19 |
| 10041 | cyp19a1: Aromatase (Anguilla japonica) | 0.89162 | 0.99998 | 0.33143 | 0.05283 | 0.99958 | 0.47739 | -0.08 | -0.11 | -0.26 | 0.59 | -0.52 | -0.10 |
| 13334 | esr2a: Estrogen receptor beta-1 (Carassius auratus) | 0.60153 | 0.99998 | 0.24249 | 0.07156 | 0.99958 | 0.52912 | 0.17 | 0.11 | -0.24 | 0.28 | -0.07 | 0.02 |
| 20836 | esr2: Estrogen receptor beta (Micropogonias undulatus) | 0.85579 | 0.99998 | 0.30342 | 0.17659 | 0.99958 | 0.20425 | -0.15 | 0.03 | -0.41 | -0.38 | -0.35 | 0.54 |
| 36485 | esr2: Estrogen receptor beta (Micropogonias undulatus) | 0.42711 | 0.99998 | 0.23652 | 0.04822 | 0.99958 | 0.07218 | 0.50 | 0.29 | -0.62 | 0.79 | -0.18 | -0.68 |
| 4717 | EPS8L2: Epidermal growth factor receptor kinase substrate 8-like protein 2 (Pongo abelii) | 0.80481 | 0.99998 | 0.28979 | 0.26840 | 0.99958 | 0.31166 | -0.40 | -0.10 | 0.79 | -0.43 | 0.29 | 0.57 |
| 6593 | EPS8L1: Epidermal growth factor receptor kinase substrate 8-like protein 1 (Homo sapiens) | 0.89314 | 0.99998 | 0.23704 | 0.05465 | 0.99958 | 0.09590 | -0.16 | 0.66 | -0.84 | 1.04 | -0.13 | -1.05 |
| 6890 | EPS15L1: Epidermal growth factor receptor substrate 15-like 1 (Homo sapiens) | 0.89821 | 0.99998 | 0.32270 | 0.05783 | 0.99958 | 0.29960 | 0.08 | -0.03 | 0.31 | -0.67 | 0.16 | 0.29 |
| 7638 | EPS15: Epidermal growth factor receptor substrate 15 (Homo sapiens) | 0.90281 | 0.99998 | 0.33262 | 0.14440 | 0.99958 | 0.21055 | -0.09 | -0.24 | -0.30 | -0.39 | 0.16 | 0.37 |
| 16729 | Eps8: Epidermal growth factor receptor kinase substrate 8 (Rattus norvegicus) | 0.82077 | 0.99998 | 0.23652 | 0.04631 | 0.99958 | 0.35057 | -0.17 | 0.24 | -0.54 | 0.70 | 0.16 | -0.19 |
| 19135 | EPS8L1: Epidermal growth factor receptor kinase substrate 8-like protein 1 (Homo sapiens) | 0.79828 | 0.99998 | 0.23652 | 0.41074 | 0.99958 | 0.33368 | -0.14 | -0.08 | 0.42 | 0.01 | -0.52 | 0.20 |
| 20203 | Egfr: Epidermal growth factor receptor (Mus musculus) | 0.91881 | 0.99998 | 0.23652 | 0.12775 | 0.99958 | 0.19060 | 0.01 | -0.34 | 1.09 | -0.70 | -0.43 | 0.74 |
| 24583 | EPS8L3: Epidermal growth factor receptor kinase substrate 8-like protein 3 (Homo sapiens) | 0.73167 | 0.99998 | 0.51144 | 0.12304 | 0.99958 | 0.19411 | 0.40 | 0.50 | -0.22 | 1.18 | -1.22 | -1.65 |
| 28582 | EPS15L1: Epidermal growth factor receptor substrate 15-like 1 (Homo sapiens) | 0.87100 | 0.99998 | 0.26689 | 0.04740 | 0.99958 | 0.14422 | -0.08 | -0.07 | 0.26 | -0.49 | 0.02 | 0.33 |
| 32174 | EPS15: Epidermal growth factor receptor substrate 15 (Homo sapiens) | 0.89297 | 0.99998 | 0.31915 | 0.25026 | 0.99958 | 0.54684 | -0.09 | -0.15 | 0.34 | -0.24 | 0.19 | -0.01 |
| 32725 | EPS15L1: Epidermal growth factor receptor substrate 15-like 1 (Homo sapiens) | 0.72659 | 0.99998 | 0.24648 | 0.04997 | 0.99958 | 0.25908 | -0.25 | -0.11 | 0.48 | -0.76 | 0.18 | 0.36 |
| 35679 | EPS8L2: Epidermal growth factor receptor kinase substrate 8-like protein 2 (Homo sapiens) | 0.87757 | 0.99998 | 0.23704 | 0.05716 | 0.99958 | 0.27663 | -0.38 | -0.23 | 1.71 | 2.13 | -1.11 | -1.19 |
| 36953 | EPS15: Epidermal growth factor receptor substrate 15 (Homo sapiens) | 0.73396 | 0.99998 | 0.28039 | 0.15125 | 0.99958 | 0.44222 | 0.14 | -0.27 | 0.21 | 0.21 | 0.19 | -0.09 |
| 38148 | EPS8: Epidermal growth factor receptor kinase substrate 8 (Pongo abelii) | 0.65586 | 0.74117 | 0.54950 | 0.41600 | 0.99958 | 0.46168 | -0.44 | -1.02 | 0.05 | -0.01 | -0.02 | -0.17 |
| 41122 | EPS8: Epidermal growth factor receptor kinase substrate 8 (Homo sapiens) | 0.30545 | 0.99998 | 0.23704 | 0.15465 | 0.99958 | 0.07846 | 0.34 | 0.24 | -0.33 | 0.22 | -0.05 | 0.40 |
| 42209 | Egfr: Epidermal growth factor receptor (Mus musculus) | 0.82435 | 0.99998 | 0.55636 | 0.08219 | 0.99958 | 0.27345 | 0.13 | -0.18 | 0.01 | 0.42 | -0.62 | -0.25 |
| 44328 | EPS15L1: Epidermal growth factor receptor substrate 15-like 1 (Homo sapiens) | 0.91334 | 0.99998 | 0.25804 | 0.04916 | 0.99958 | 0.24035 | -0.04 | 0.01 | 0.63 | -1.13 | 0.28 | 0.53 |
| 45179 | Egfr: Epidermal growth factor receptor (Mus musculus) | 0.90334 | 0.99998 | 0.43323 | 0.39996 | 0.96113 | 0.07756 | -0.04 | -0.20 | 0.12 | -0.02 | -0.75 | 0.52 |
| 34828 | FSHR: Follicle-stimulating hormone receptor (Cairina moschata) | 0.43019 | 0.99998 | 0.23652 | 0.34037 | 0.99958 | 0.39662 | -1.14 | 0.48 | -1.04 | 0.19 | -0.66 | 0.36 |

**Table S6.** Likelihood ratio test on rate of undifferentiated gonads explained by time point of sampling and exposure to ethinylestradiol (EE2), the bacterium *P. fluorescens* (PF), or both (EE2 x PF), N_total_ = 249. This analysis is based on the assumption that the observed contamination of probes (see main text) happened after sampling from the control aquaria.

| Effect | χ^2^ | d.f. | P |
| --- | --- | --- | --- |
| **Time** | **72.5** | **4** | **<0.0001** |
| EE2 | <0.1 | 1 | 1.0 |
| PF | <0.1 | 1 | 1.0 |
| **Time x EE2** | **9.7** | **4** | **0.045** |
| Time x PF | 0.9 | 4 | 0.93 |
| EE2 x PF | <0.1 | 1 | 1.0 |
| Time x EE2 x PF | 4.6 | 4 | 0.33 |

**Table S7.** RNA quality and RNA-sequencing. ID: sample id; TP: time point (1 = embryo stage, 2 = hatchling stage, 3 = first feeding stage); TR: treatment: SI: sibgroup; Sex: PCR-determined genetic sex; 260/280 and 260/230: absorbance ratios (Nanodrop); ng/ul (FA): fragment analyzer concentration of RNA; LA: sequencing lane; YI: sequencing yield in Mbases; RR: number of raw reads; QS: mean reads quality score from Illumina. Adapted from Maitre et al., 2017.

| **ID** | **TP** | **TR** | **SI** | **Sex** | **260/280** | **260/230** | **ng/ul (FA)** | **RQN** | **LA** | **YI** | **RR** | **QS** |
| --- | --- | --- | --- | --- | --- | --- | --- | --- | --- | --- | --- | --- |
| 1 | 1 | control | 93 | M | 2.12 | 1.96 | 64.56 | 9.7 | D | 8951 | 94,310,746 | 35.23 |
| 2 | 1 | control | 94 | M | 2.34 | 1.69 | 53.2 | 10 | H | 7728 | 80,727,600 | 35.32 |
| 3 | 1 | control | 95 | M | 1.98 | 1.13 | 38.85 | 9 | D | 8434 | 89,100,552 | 35.22 |
| 4 | 1 | control | 96 | M | 2.05 | 1.73 | 86.4 | 8.6 | F | 7202 | 74,249,340 | 35.51 |
| 5 | 1 | control | 97 | M | 2.14 | 2.1 | 108.29 | 9.8 | C | 9339 | 98,912,292 | 35.03 |
| 6 | 1 | EE2 | 93 | M | 2.05 | 2.36 | 69.83 | 10 | F | 7986 | 84,077,272 | 35.08 |
| 8 | 1 | EE2 | 95 | M | 2.12 | 2 | 79.97 | 9.1 | B | 9476 | 100,430,984 | 35.08 |
| 9 | 1 | EE2 | 96 | M | 2.08 | 2.09 | 51.4 | 9.7 | G | 7159 | 73,602,988 | 35.74 |
| 12 | 2 | control | 94 | F | 1.92 | 1.33 | 40.3 | 9.2 | I | 8590 | 88,975,372 | 35.49 |
| 13 | 2 | control | 95 | M | 2.1 | 2.25 | 56.93 | 9.5 | L | 8373 | 87,518,388 | 35.27 |
| 14 | 2 | control | 96 | M | 2.37 | 2.76 | 36.99 | 10 | G | 5453 | 57,482,172 | 34.73 |
| 15 | 2 | control | 97 | M | 2.04 | 1.17 | 35.56 | 9 | B | 8376 | 89,584,502 | 34.81 |
| 16 | 2 | EE2 | 93 | M | 2.12 | 2.61 | 63.84 | 9.5 | E | 6814 | 72,385,354 | 34.85 |
| 18 | 2 | EE2 | 95 | M | 2.07 | 1.07 | 57.25 | 8.5 | G | 9822 | 101,412,350 | 35.5 |
| 19 | 2 | EE2 | 96 | M | 2.2 | 2.64 | 45.94 | 9.9 | A | 8864 | 93,949,962 | 35.2 |
| 20 | 2 | EE2 | 97 | M | 2.15 | 2.44 | 48.54 | 9.1 | C | 9948 | 105,392,198 | 35.04 |
| 21 | 3 | control | 93 | M | 2.07 | 1.6 | 55.83 | 8.1 | A | 8917 | 94,149,480 | 35.19 |
| 22 | 3 | control | 94 | M | 2.11 | 1.85 | 74.5 | 8.3 | I | 8797 | 91,463,958 | 35.25 |
| 23 | 3 | control | 95 | M | 2.16 | 2.61 | 115.08 | 8.1 | I | 10727 | 111,651,906 | 35.36 |
| 24 | 3 | control | 96 | M | 2.09 | 1.48 | 68.65 | 7.8 | I | 6311 | 65,582,824 | 35.33 |
| 25 | 3 | control | 97* | M | 2.13 | 1.8 | 202.69 | 8.7 | L | 10682 | 111,546,226 | 35.21 |
| 26 | 3 | EE2 | 93 | M | 2.15 | 2.18 | 74.68 | 8.4 | L | 4978 | 52,219,004 | 35.1 |
| 27 | 3 | EE2 | 94 | M | 2.1 | 2.42 | 86.62 | 9.2 | E | 10277 | 108,106,904 | 35.06 |
| 28 | 3 | EE2 | 95 | M | 2.03 | 2 | 58.83 | 8 | H | 8803 | 92,053,888 | 35.19 |
| 30 | 3 | EE2 | 97* | M | 2.09 | 1.98 | 145.31 | 9.1 | G | 6081 | 62,680,856 | 35.38 |
| 31 | 1 | control | 93 | F | 1.84 | 0.98 | 40.86 | 9.5 | I | 7467 | 77,588,734 | 35.27 |
| 33 | 1 | control | 95 | F | 2.12 | 1.63 | 63.04 | 9.5 | H | 8544 | 89,545,780 | 35.28 |
| 34 | 1 | control | 96 | F | 2.25 | 2.13 | 65.33 | 8.6 | E | 8823 | 92,375,346 | 35.23 |
| 35 | 1 | control | 97 | F | 2.08 | 1.84 | 98.04 | 9.8 | C | 8618 | 91,765,202 | 34.81 |
| 36 | 1 | EE2 | 93 | F | 2.22 | 1.48 | 49.47 | 9.7 | F | 10423 | 110,533,770 | 34.97 |
| 37 | 1 | EE2 | 94 | M | 2.11 | 1.98 | 88.29 | 9.7 | G | 7630 | 81,074,258 | 34.91 |
| 38 | 1 | EE2 | 95 | F | 2.08 | 1.78 | 69.2 | 10 | I | 7912 | 82,886,272 | 35.05 |
| 39 | 1 | EE2 | 96 | F | 2.16 | 1.68 | 78.38 | 9.1 | B | 7781 | 83,083,296 | 34.99 |
| 41 | 2 | control | 93 | F | 2.05 | 1.27 | 63.17 | 9 | F | 9518 | 100,729,174 | 35.13 |
| 42 | 2 | control | 94 | F | 2.04 | 1.83 | 45.53 | 8.9 | B | 8168 | 86,722,276 | 35.14 |
| 43 | 2 | control | 95 | F | 2.02 | 2.26 | 44.24 | 9.4 | B | 9226 | 97,628,958 | 35.13 |
| 44 | 2 | control | 96 | F | 2.16 | 1.71 | 38.48 | 10 | D | 9512 | 100,112,566 | 34.99 |
| 45 | 2 | control | 97 | F | 2.22 | 1.88 | 32.23 | 10 | L | 8145 | 85,152,984 | 35.28 |
| 46 | 2 | EE2 | 93 | F | 1.97 | 2.36 | 65.55 | 9.9 | E | 9012 | 95,051,902 | 35.03 |
| 47 | 2 | EE2 | 94 | F | 2.19 | 1.65 | 60.85 | 9.1 | A | 8993 | 95,242,488 | 35.12 |
| 48 | 2 | EE2 | 95 | F | 2.09 | 2.4 | 58.88 | 9.2 | C | 9169 | 96,915,018 | 34.94 |
| 49 | 2 | EE2 | 96 | F | 2.05 | 2.18 | 48.67 | 10 | H | 7839 | 82,485,106 | 35 |
| 50 | 2 | EE2 | 97 | F | 2.08 | 2.01 | 59.54 | 8.7 | D | 8872 | 93,464,052 | 35.11 |
| 51 | 3 | control | 93 | F | 2.14 | 1.96 | 52.75 | 9.8 | D | 8775 | 92,371,168 | 35.05 |
| 52 | 3 | control | 94 | F | 2.12 | 1.45 | 81.82 | 8.8 | C | 8920 | 94,572,332 | 34.89 |
| 53 | 3 | control | 95 | F | 2.14 | 2.04 | 96.53 | 8.3 | E | 10503 | 110,538,692 | 35.04 |
| 54 | 3 | control | 96 | F | 2.14 | 1.84 | 64.31 | 7.2 | G | 9787 | 100,738,916 | 35.45 |
| 55 | 3 | control | 97* | M | 2.13 | 2.15 | 172.89 | 8.3 | C | 8451 | 89,566,368 | 34.97 |
| 56 | 3 | EE2 | 93 | M | 2.11 | 2.27 | 132.32 | 8 | A | 8874 | 94,121,488 | 35.02 |
| 57 | 3 | EE2 | 94 | F | 2.03 | 1.55 | 94.42 | 8.5 | E | 9180 | 96,847,664 | 35.12 |
| 58 | 3 | EE2 | 95 | F | 2.04 | 2.62 | 80.9 | 6.1 | D | 8642 | 90,872,204 | 35.25 |
| 59 | 3 | EE2 | 96 | F | 1.9 | 1.53 | 57.42 | 8.9 | A | 9067 | 95,871,206 | 35.26 |
| 60 | 3 | EE2 | 97* | F | 2.13 | 1.86 | 130.93 | 8.2 | H | 9946 | 106,909,132 | 34.43 |
| 61 (29) | 3 | EE2 | 96 | M | 1.99 | 3.04 | 79.19 | 8.5 | L | 9395 | 98,030,932 | 35.22 |
| 63 (40) | 1 | EE2 | 97 | F | 2.06 | 1.8 | 101.32 | 9.3 | H | 10255 | 107,142,268 | 35.3 |
| 64 (32) | 1 | control | 94 | F | 1.98 | 1.41 | 87.3 | 9.6 | L | 9710 | 103,383,790 | 34.82 |
| 65(10) | 1 | EE2 | 97 | M | 2.12 | 1.45 | 122.38 | 9.9 | F | 5254 | 55,447,016 | 35.35 |
| 66(11) | 2 | control | 93 | M | 2.02 | 0.9 | 78.83 | 8.9 | A | 8567 | 90,719,714 | 35.01 |
| 67(17) | 2 | EE2 | 94 | M | 2 | 1.3 | 48 | 7 | F | 8300 | 89,764,786 | 34.51 |
| 68(37) | 1 | EE2 | 94 | M | 2.02 | 1.2 | 59.4 | 7.5 | B | 9629 | 101,343,034 | 35.04 |
